# FAR591 promotes the pathogenesis and progression of SONFH by regulating Fos expression to mediate the apoptosis of bone microvascular endothelial cells

**DOI:** 10.1038/s41413-023-00259-8

**Published:** 2023-05-22

**Authors:** Fei Zhang, Lei Wei, Lei Wang, Tao Wang, Zhihong Xie, Hong Luo, Fanchao Li, Jian Zhang, Wentao Dong, Gang Liu, Qinglin Kang, Xuesong Zhu, Wuxun Peng

**Affiliations:** 1 Department of Emergency Orthopedics, The Affiliated Hospital of Guizhou Medical University, Guiyang, Guizhou 550004 China; 2 School of Clinical Medicine, Guizhou Medical University, Guiyang, Guizhou 550004 China; 3 Department of Orthopedics, Rhode Island Hospital, Brown University, Providence, Rhode Island 02903 USA; 4 Department of Orthopedics, Shanghai Jiao Tong University Affiliated Sixth People’s Hospital, Shanghai, 200233 China; 5 Department of Orthopedics, The First Affiliated Hospital of Soochow University, Suzhou, Jiangsu 215000 China

**Keywords:** Bone, Pathogenesis

## Abstract

The specific pathogenesis of steroid-induced osteonecrosis of the femoral head (SONFH) is still not fully understood, and there is currently no effective early cure. Understanding the role and mechanism of long noncoding RNAs (lncRNAs) in the pathogenesis of SONFH will help reveal the pathogenesis of SONFH and provide new targets for its early prevention and treatment. In this study, we first confirmed that glucocorticoid (GC)-induced apoptosis of bone microvascular endothelial cells (BMECs) is a pre-event in the pathogenesis and progression of SONFH. Then, we identified a new lncRNA in BMECs via lncRNA/mRNA microarray, termed Fos-associated lincRNA ENSRNOT00000088059.1 (FAR591). FAR591 is highly expressed during GC-induced BMEC apoptosis and femoral head necrosis. Knockout of FAR591 effectively blocked the GC-induced apoptosis of BMECs, which then alleviated the damage of GCs to the femoral head microcirculation and inhibited the pathogenesis and progression of SONFH. In contrast, overexpression of FAR591 significantly promoted the GC-induced apoptosis of BMECs, which then aggravated the damage of GCs to the femoral head microcirculation and promoted the pathogenesis and progression of SONFH. Mechanistically, GCs activate the glucocorticoid receptor, which translocates to the nucleus and directly acts on the FAR591 gene promoter to induce FAR591 gene overexpression. Subsequently, FAR591 binds to the Fos gene promoter (–245∼–51) to form a stable RNA:DNA triplet structure and then recruits TATA-box binding protein associated factor 15 and RNA polymerase II to promote Fos expression through transcriptional activation. Fos activates the mitochondrial apoptotic pathway by regulating the expression of Bcl-2 interacting mediator of cell death (Bim) and P53 upregulated modulator of apoptosis (Puma) to mediate GC-induced apoptosis of BMECs, which leads to femoral head microcirculation dysfunction and femoral head necrosis. In conclusion, these results confirm the mechanistic link between lncRNAs and the pathogenesis of SONFH, which helps reveal the pathogenesis of SONFH and provides a new target for the early prevention and treatment of SONFH.

## Introduction

Steroid-induced osteonecrosis of the femoral head (SONFH) is a joint dysfunction disease caused by long-term and high-dose use of glucocorticoids (GCs).^1,2^ In the early stage, the main manifestations of SONFH are necrosis and cystic degeneration of the femoral head, while in the late stage, the collapse of the femoral head seriously affects the patient’s function and quality of life.^3,4^ Recently, with the widespread use of GCs, the incidence of SONFH has increased annually, ranking first among nontraumatic femoral head necrosis. If SONFH is not treated in a timely manner, the rate of collapse of the femoral head within 3 years is more than 80%, with a high disability rate; thus, early prevention and treatment are crucial to prevent long-term disability.^5,6^ However, as the specific pathogenesis of SONFH is still unclear, existing treatments remain unable to definitively cure SONFH in the early stage.^7^ Therefore, an in-depth study of the pathogenesis of SONFH has important practical value for the early prevention and treatment of SONFH.

The pathogenesis of SONFH is related to bone microcirculation dysfunction and osteogenic damage.^8,9^ Bone microcirculation and osteogenesis are coupled with each other via bone microvascular endothelial cells (BMECs). BMECs not only secrete vasodilators, anticoagulants, and vascular endothelial growth factors to regulate microvascular relaxation, inhibit thrombosis, and promote vascular regeneration to maintain the stability of bone microcirculation but also secrete basic fibroblast growth factor, transforming growth factor, noggin, bone morphogenetic protein, and other osteogenic regulatory factors to promote the proliferation and differentiation of osteoblasts (OBs) and their progenitor cells to initiate bone regeneration.^10–16^ However, in the process of GC-induced femoral head necrosis, GCs can induce BMEC apoptosis and inhibit vascular regeneration, resulting in bone microcirculation dysfunction, which will interrupt the coupling mechanism of bone microcirculation and osteogenesis, resulting in osteogenic damage and osteonecrosis.^17–22^ Therefore, studying the molecular events of GC-induced apoptosis of BMECs will help to reveal the pathogenesis of SONFH and provide new targets for its prevention and treatment.

Studies have shown that the mitochondrial apoptotic pathway can respond to glucocorticoid receptor (GR)-mediated upstream apoptotic signals and play a role in amplifying and executing apoptotic signals in GC-induced apoptosis.^23–25^ For example, GCs can induce the downregulation of antiapoptotic proteins, such as B-cell lymphoma 2 (Bcl-2), B-cell lymphoma/leukemia-xL (Bcl-xL), and myeloid cell leukemia-1, and can induce the upregulation of proapoptotic proteins, such as Bcl-2 interacting mediator of cell death (Bim), P53 upregulated modulator of apoptosis (Puma), Bcl-2 modifying factor (Bmf), Bcl-xL/Bcl-2-associated death promoter, Bcl-2 associated X protein, and caspase 3 (CASP3), which are the main regulators and executors of the mitochondrial apoptotic pathway.^26–28^ However, the upstream regulatory mechanism underlying the abnormal expression of these genes in the process of GC-induced apoptosis of BMECs is poorly understood.

Long noncoding RNAs (lncRNAs) are important regulators of gene expression at the transcriptional level (such as controlling transcription factors, histone modification, DNA methylation, enhancer activity, and transcription interference), post-transcriptional level (such as RNA methylation, RNA alternative splicing, and RNA stability), translational level (such as adjustment of translation initiation and extension), and post-translational level (such as ubiquitination and phosphorylation of protein). As a result, lncRNAs intervene in cell apoptosis, differentiation, and proliferation and play a role in the pathogenesis and progression of various diseases.^29–42^ However, it remains unclear whether lncRNAs are involved in regulating the aberrant expression of mitochondrial apoptotic pathway genes, which in turn mediates BMEC apoptosis and the pathogenesis of SONFH.

In this study, we identified a new lncRNA in BMECs using lncRNA/mRNA microarray analysis and defined it as Fos-associated lincRNA ENSRNOT00000088059.1 (FAR591) according to the HGNC nomenclature guidelines, as well as its genomic location and molecular function. FAR591 is related to the abnormal expression of mitochondrial apoptotic pathway genes and is significantly overexpressed in the processes of GC-induced BMEC apoptosis and femoral head necrosis. Therefore, this study further revealed the role and regulatory mechanism of FAR591 in GC-induced BMEC apoptosis and femoral head necrosis.

## Results

### GC-induced BMEC apoptosis is a pre-event in the pathogenesis and progression of SONFH

**Fig. 1:**
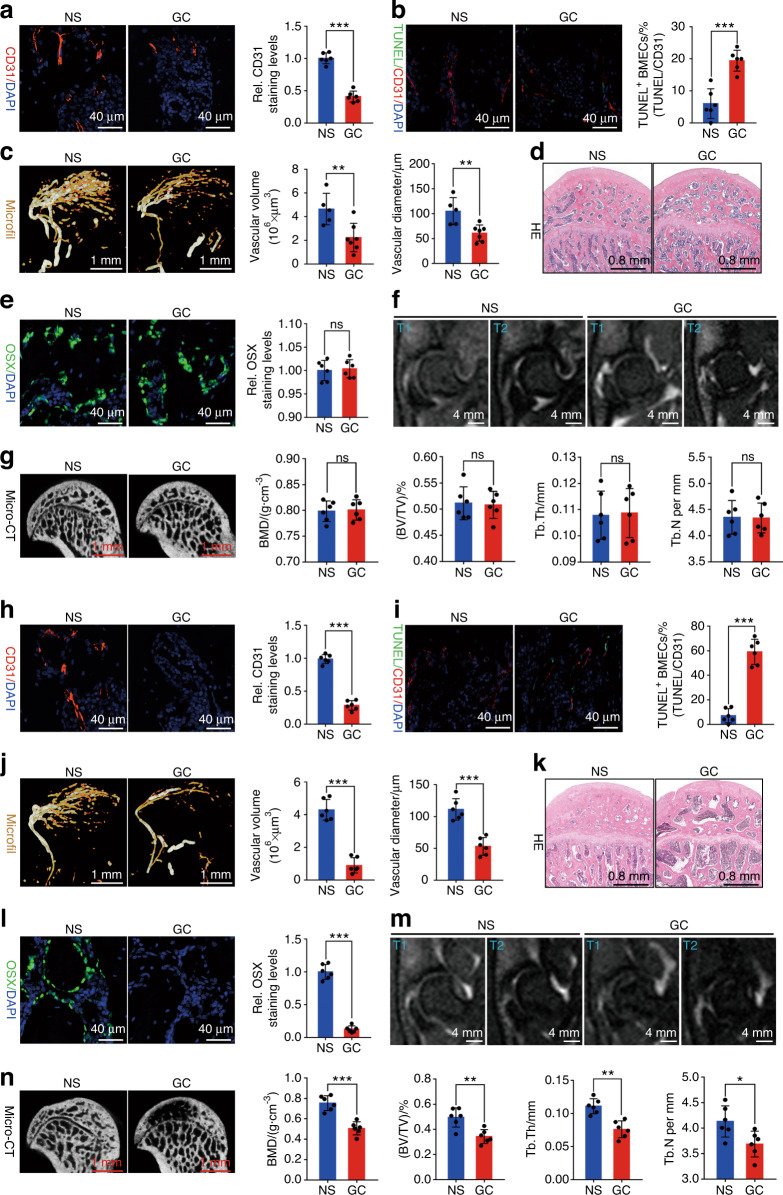
GC-induced BMEC apoptosis is a pre-event in the pathogenesis and progression of SONFH. MP was used to establish the SONFH model (MP is a GC used for intramuscular injection) 2 weeks after MP gluteal injection: **a** CD31 immunostaining level in BMECs (*n* = 6). **b** Proportion of TUNEL^+^ cells in CD31^+^ BMECs (*n* = 6). **c** Microfil angiography was used to detect the microvessel volume and blood flow (*n* = 6). **d** HE staining was used to evaluate osteonecrosis (*n* = 6). **e** OSX immunostaining level in OBs (*n* = 6). MRI (**f**) and micro-CT (**g**) evaluation of femoral head necrosis (*n* = 6). Four weeks after MP injection: **h** CD31 immunostaining level in BMECs (*n* = 6). **i** Proportion of TUNEL^+^ cells in CD31^+^ BMECs (*n* = 6). **j** Microfil angiography was used to detect the microvessel volume and blood flow (*n* = 6). **k** HE staining was used to evaluate osteonecrosis (*n* = 6). **l** OSX immunostaining levels in OBs (*n* = 6). MRI (**m**) and micro-CT (**n**) evaluation of femoral head necrosis (*n* = 6). In (**a–c**, **e**, **g**, **h–j**, **l**, **n**), data are presented as the means ± standard deviations (SDs); statistical significance was calculated by Student’s *t* test; ^*^P < 0.05, ^**^P < 0.01, ^***^P < 0.001. Abbreviations: glucocorticoid (GC), bone microvascular endothelial cell (BMEC), steroid-induced osteonecrosis of the femoral head (SONFH), methylprednisolone (MP), normal saline (NS), 4’,6-diamidino-2-phenylindole (DAPI), TdT-mediated dUTP nick-end labeling (TUNEL), Osterix (OSX), magnetic resonance imaging (MRI), bone mineral density (BMD), tissue volume (TV), bone volume (BV), trabecular number (Tb. N), and trabecular thickness (Tb. Th)

The impaired coupling mechanism of bone microcirculation and osteogenesis is the core event of GC-induced femoral head necrosis, but the pre-event of damage to the coupling mechanism remains controversial.^43–48^ BMECs and OBs are the main functional cells of angiogenesis and osteogenesis, respectively. We tested the toxicity of hydrocortisone (HC; the active form of GCs) to BMECs and OBs. The results showed that BMECs were more sensitive to HC, and a low concentration of HC could induce high rates of BMEC apoptosis and inhibit their angiogenesis (Fig. S1), while higher concentrations of HC were needed to induce OB apoptosis and inhibit its osteogenesis (Fig. S2). We also used methylprednisolone (MP; a GC commonly used for intramuscular injection) to induce femoral head necrosis in Sprague‒Dawley (SD) rats. At different time points (2 weeks and 4 weeks) after MP injection, we detected the apoptosis of femoral head BMECs, the volume of microvessels, the expression level of osteogenic markers, and the progress of femoral head necrosis. Two weeks after MP injection, the expression level of CD31 in the femoral head decreased (Fig. 1a), the rate of CD31^+^-TUNEL^+^ double-positive cells increased significantly (Fig. 1b), and angiography showed that both the microvessel volume and blood flow in the subchondral area of the femoral head decreased (Fig. 1c). However, there was no significant change in the expression level of the osteogenic marker osterix (OSX; Fig. 1e). HE staining showed that the trabeculae were normal, with clusters of OBs surrounding them, and there were no signs of osteonecrosis, such as empty bone lacunae (Fig. 1d). Magnetic resonance imaging (MRI; T1- and T2-weighted images [WI]) showed no abnormal signals in the femoral head (Fig. 1f). Micro-CT showed that the shape of the femoral head and the arrangement of the subchondral trabeculae were normal without thinning and interruption, and there were no significant changes in bone mineral density (BMD), bone volume fraction (BVF), trabecular number (TB. N), and trabecular thickness (TB. Th) (Fig. 1g). Moreover, TRAP staining showed no significant change in the number of osteoclasts or bone resorptive activity (Fig. S3A–D). Four weeks after MP injection, the expression level of CD31 in the femoral head continued to decrease (Fig. 1h), the rate of CD31^+^-TUNEL^+^ double-positive cells continued to increase (Fig. 1I), and angiography showed that the microvessel volume and blood flow in the subchondral area of the femoral head further decreased (Fig. 1j). At this time, the expression level of OSX decreased (Fig. 1l). HE staining showed many empty bone lacunae in the bone trabeculae, as well as disappearance of the surrounding osteoblasts (Fig. 1k). MRI showed an abnormally high signal in the subchondral area of the femoral head on T2-WI and a low signal on T1-WI (Fig. 1m). Micro-CT showed disorder, thinning, and interruption of the subchondral trabecula, as well as significant decreases in BMD, BVF, Tb. N, and TB. Th (Fig. 1n). TRAP staining showed an increase in the osteoclast number and bone resorptive activity (Fig. S3E–H). These results showed that during GC-induced femoral head necrosis, BMEC apoptosis and microcirculation dysfunction occurred first, followed by osteogenic damage, osteonecrosis, and increased bone resorption. Therefore, an in-depth study on the mechanism of GC-induced apoptosis of BMECs is essential to reveal the pathogenesis of SONFH.

### FAR591 was associated with GC-induced apoptosis of BMECs and necrosis of the femoral head

**Fig. 2:**
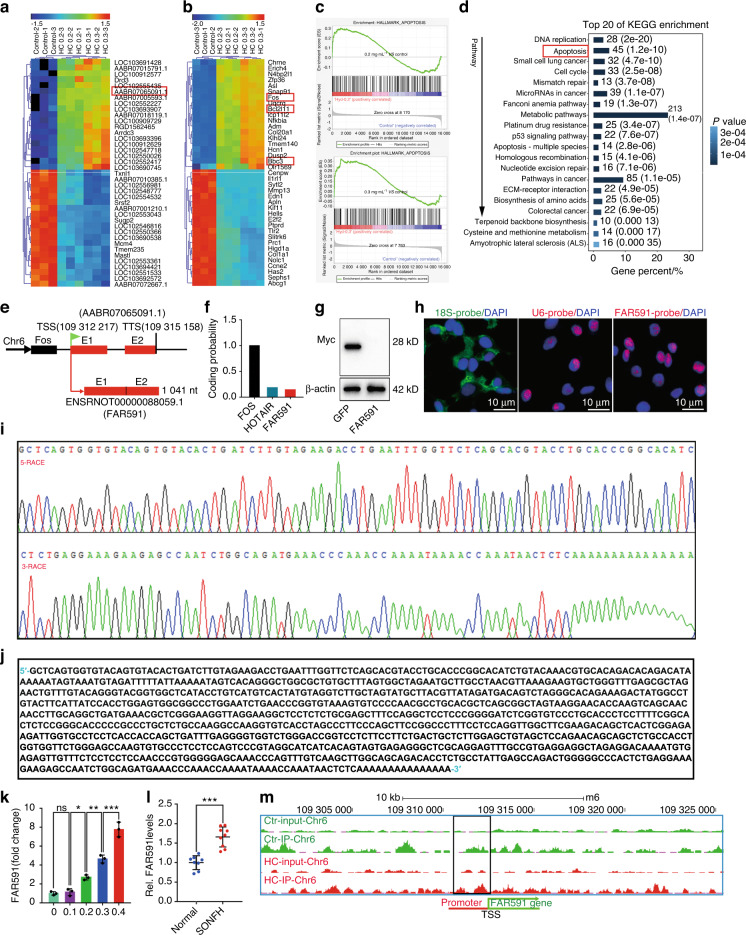
FAR591 is related to GC-induced BMEC apoptosis and femoral head necrosis. **a** LncRNA cluster analysis. **b** mRNA cluster analysis. **c** Apoptotic GSEA. **d** KEGG enrichment analysis showed that the apoptotic signaling pathway in the red box was significantly enriched. **e** Localization of FAR591 in the genome and transcript information. **f** CPC software predicted the protein coding potential of FAR591. **g** The expression plasmid pcDNA4/myc-His was used to verify the protein coding ability of FAR591 (*n* = 3), and green fluorescent protein (GFP) was used as the positive control of the coding protein. **h** Subcellular localization of FAR591 was detected by RNA-FISH (*n* = 4). **i** Peak diagram of the full-length sequence of FAR591 detected by RACE (*n* = 3). **j** Splicing results of the full-length sequence of FAR591. **k** Validation of FAR591 expression in the model of BMEC apoptosis induced by different concentrations of HC (*n* = 3). **l** The expression of FAR591 was detected in BMECs of SONFH tissue (*n* = 9). **m** Visualized results of GR enrichment on chromosome 6 (FAR591 promoter region) (*n* = 3). In (**k, l**), data are presented as the means ± SDs; statistical significance was calculated by one-way ANOVA with Tukey’s *post hoc* tests (**k**) and Student’s t tests (**l**); ^*^P < 0.05, ^**^P < 0.01, ^***^P < 0.001. Abbreviations: glucocorticoid receptor (GR), long noncoding RNA (lncRNA), messenger RNA (mRNA), gene set enrichment analysis (GSEA), Kyoto Encyclopedia of Genes and Genomes (KEGG), transcription start site (TSS), transcription termination site (TTS), exon 1 (E1), exon 2 (E2), fluorescence in situ hybridization (FISH)

GC-induced apoptosis is related to abnormal expression of mitochondrial apoptotic pathway gene clusters.^23–25^ LncRNAs are important regulators of gene expression.^49–56^ It is unclear whether lncRNAs are involved in regulating the abnormal expression of mitochondrial apoptotic pathway gene clusters before mediating GC-induced BMEC apoptosis. We screened 105 GC concentration-dependent lncRNAs (46 upregulated and 59 downregulated) and 345 GC concentration-dependent mRNAs (153 upregulated and 192 downregulated) by lncRNA/mRNA microarray analysis in the model of BMEC apoptosis induced by different concentrations of GCs (fold change > 2, *P* < 0.05; Fig. 2a, b). The verification data of the microarray are shown in Fig. S4A–H. Kyoto Encyclopedia of Genes and Genomes (KEGG) and gene set enrichment analysis (GSEA) showed that GC concentration-dependent mRNAs were significantly enriched in the apoptotic signal pathway, and the mRNAs enriched in the apoptotic signal pathway were mainly related to the mitochondrial apoptotic pathway (*P* < 0.05; Fig. 2c, d). We selected the core mRNAs of the mitochondrial apoptotic pathway from the GC concentration-dependent mRNAs and then identified the apoptosis-related candidate lncRNAs by mRNA‒lncRNA coexpression analysis (Pearson correlation coefficient > 0.99, *P* < 0.01) and adjacent gene screening (distance <200 kb; Fig. S4I–J). The transcript ID of this candidate lncRNA was ENSRNOT00000088059.1 (Fig. S4K). Rapid amplification of the cDNA end (RACE) showed that the full-length sequence of this transcript was 1 041 nt (Fig. 2i, j). The alignment of the sequence with the genome showed that the GeneSymbol corresponding to this transcript was AABR07065091.1, and the locus was located on chromosome 6 (109 312 217–109 315 245), which consisted of two exons and one intron and was downstream of the Fos gene, which was consistent with the transcription direction of Fos (Fig. 2e). The CPC^57^ (http://cpc.cbi.pku.edu.cn) predicted that this transcript had no ability to encode protein using HOX transcript antisense RNA as a negative control for noncoding genes and Fos as a positive control for coding genes (Fig. 2f). Then, the transcript was cloned into a pcDNA4/myc-His expression plasmid and transfected into BMECs. The expression of the myc fusion protein was analyzed by immunoblotting with an anti-myc antibody. We confirmed that the transcript could not encode protein, and GFP was used as the positive control of the encoded protein (Fig. 2g). RNA fluorescence in situ hybridization (FISH) showed that the transcript was mainly located in the nucleus, with 18S and U6 used as positive controls for cytoplasmic and nuclear RNA, respectively (Fig. 2h). According to the guidelines for lncRNA nomenclature provided by the HUGO Gene Nomenclature Committee,^58^ we defined this transcript as a Fos-associated lincRNA ENSRNOT00000088059.1 (FAR591). The expression of FAR591 was verified in a model of GC-induced BMEC apoptosis, and the results showed that the expression of FAR591 was continuously upregulated with increasing GC concentration and apoptotic rate (Fig. 2k). Simultaneously, we detected significantly increased expression of FAR591 in the BMECs of SONFH tissue (Fig. 2l). The ChIP-seq data of the GR showed that it was enriched in the FAR591 promoter and that GCs promoted this process. The visual result of GR enrichment on the FAR591 gene promoter is shown in Fig. 2m. These results indicate that FAR591 is associated with GC-induced BMEC apoptosis and femoral head necrosis.

### FAR591 mediates GC-induced BMEC apoptosis

**Fig. 3:**
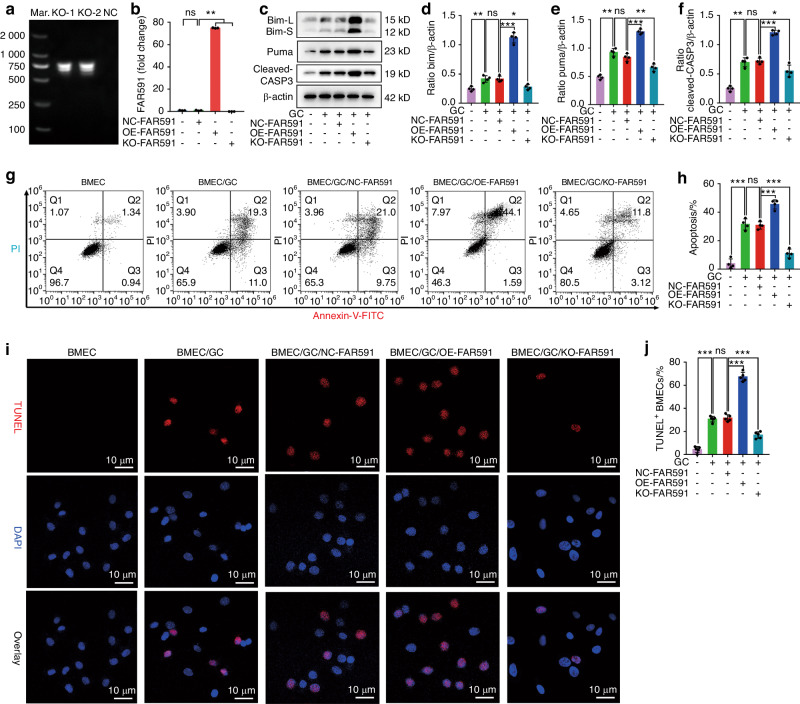
FAR591 mediated GC-induced apoptosis of BMECs. Knockout of FAR591 by CRISPR/Cas9 or upregulation of FAR591 by gene transfection overexpression: **a** Nested PCR-agarose gel electrophoresis verified FAR591 gene knockout (*n* = 3). **b** qPCR detection of FAR591 expression (*n* = 3). GC (HC)-induced apoptosis of BMECs: **c** Western blot detection of Bim, Puma, and cleaved CASP3 expression (*n* = 4). **d** Quantitative analysis of Bim expression shown in (**c**) (*n* = 4). **e** Quantitative analysis of Puma expression shown in (**c**) (*n* = 4). **f** Quantitative analysis of cleaved CASP3 expression shown in (**c**) (*n* = 4). **g**, **h** Flow cytometry was used to detect the apoptosis rate of BMECs (*n* = 4). **i**, **j** TUNEL/DAPI was used to detect the apoptosis of BMECs (n = 5). In (**b**, **d**–**f**, **h**, **j**), data are presented as the means ± SDs; statistical significance was calculated by one-way ANOVA with Tukey’s *post hoc* tests; ^*^P < 0.05, ^**^P < 0.01, ^***^P < 0.001. Abbreviations: marker (Mar.), knockout (KO), negative control (NC), overexpression of FAR591 (OE-FAR591), knockout of FAR591 (KO-FAR591), Bcl-2 interacting mediator of cell death (Bim), P53 upregulated modulator of apoptosis (Puma), caspase-3 (CASP-3), propidium iodide (PI), fluorescein isothiocyanate (FITC)

We designed four single guide RNAs for the FAR591 gene and used CRISPR/Cas9 technology to knock out the FAR591 gene in BMECs. The results of agarose gel electrophoresis of nested PCR products showed that there was no positive band in the negative control (NC) group at 500–1 000 bp, while there was an obvious positive band at 750 bp and no nonspecific amplification in the FAR591 knockout (KO-FAR591) group, which confirmed that the selected monoclonal cells were BMECs with KO-FAR591 (Fig. 3a). The qPCR results also confirmed that FAR591 was successfully knocked out in BMECs (Fig. 3b). Subsequently, we used GCs to induce BMEC apoptosis. The results showed that the expression of Bim, Puma, and cleaved CASP3 was upregulated after BMECs were treated with GCs (Fig. 3c–f), and the apoptotic rate of BMECs was increased (Fig. 3g–j). However, after the knockout of FAR591, the expression of Bim, Puma, and cleaved CASP3 was downregulated (Fig. 3c–f), and the apoptotic rate of BMECs was significantly decreased (Fig. 3g–j). These results confirmed that knockout of FAR591 blocked GC-induced apoptosis in BMECs.

We inserted the complementary deoxyribonucleic acid (cDNA) sequence of FAR591 into the overexpressed lentiviral vector (OE-FAR591) and transfected BMECs. The qPCR results confirmed that FAR591 was successfully upregulated in BMECs (Fig. 3b). Subsequently, we used GCs to induce BMEC apoptosis. The results showed that overexpression of FAR591 upregulated the expression of Bim, Puma, and cleaved CASP3 (Fig. 3c–f) and significantly increased the apoptotic rate of BMECs (Fig. 3g–j). These results confirm that overexpression of FAR591 promotes GC-induced apoptosis in BMECs.

### FAR591-mediated apoptosis of BMECs promotes the pathogenesis and progression of SONFH

**Fig. 4:**
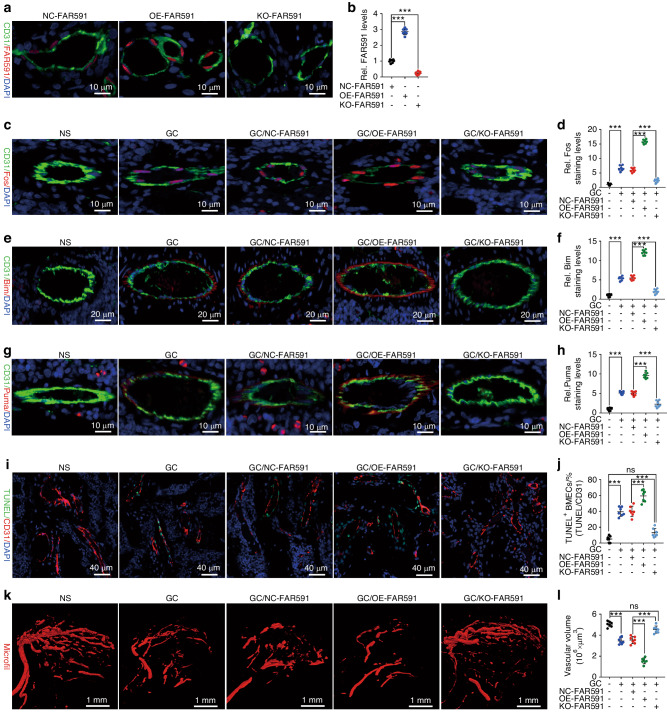
FAR591-mediated GC-induced apoptosis of BMECs impaired femoral head microcirculation. Four weeks after adeno-associated virus (serotype 9) infection in vivo: **a**, **b** The RNAscope technique was used to detect the expression level of FAR591 in BMECs (*n* = 7). After successful intervention of FAR591 expression in BMECs, GCs (MP) were used to establish the SONFH model of SD rats: **c**–**h** The immunostaining levels of Fos (**c**, **d**), Bim (**e**, **f**), and Puma (**g**, **h**) in BMECs (*n* = 7). **i**, **j** The proportion of TUNEL^+^ cells in BMECs (*n* = 7). **k**, **l** Microfil angiography detected blood flow of the femoral head (*n* = 7). In (**b**, **d**, **f**, **h**, **j**, **l**), data are presented as the means ± SDs; statistical significance was calculated by one-way ANOVA with Tukey’s *post hoc* tests; ^*^P < 0.05, ^**^P < 0.01, ^***^P < 0.001

**Fig. 5:**
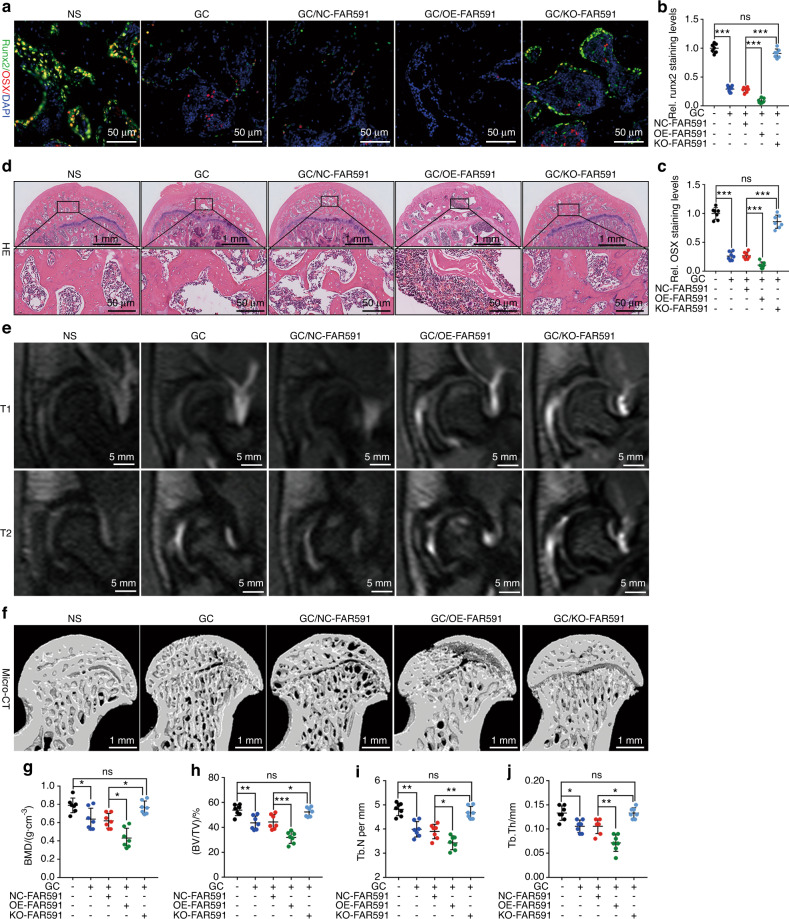
FAR591-mediated GC-induced apoptosis of BMECs promoted the pathogenesis and progression of SONFH. After FAR591 was knocked out or overexpressed in BMECs, GCs (MP) were used to establish the SONFH model in SD rats. **a–c** Immunostaining levels of Runx2 and OSX in the OBs (*n* = 7). **d** HE staining was used to assess osteonecrosis (*n* = 7). **e** MRI was used to detect femoral head necrosis (*n* = 16). **f–j** Micro-CT detected BMD, BV/TV, Tb. N, and Tb. Th in the osteonecrosis area (*n* = 7). In (**b**, **c**, **g–j**), data are presented as the means ± SDs; statistical significance was calculated by one-way ANOVA with Tukey’s *post hoc* tests; ^*^P < 0.05, ^**^P < 0.01, ^***^P < 0.001. Abbreviations: runt-related transcription factor 2 (Runx2)

To evaluate the role of FAR591 in vivo, we constructed an adeno-associated virus (serotype 9) with FAR591 gene knockout (KO-FAR591) and overexpression (OE-FAR591) and infected BMECs of SD rats in vivo. At the 4th week after adeno-associated virus infection, the expression level of FAR591 was detected using the RNAscope technique (CD31-labeled BMECs). Compared to that in the negative control group, the expression of FAR591 in the OE-FAR591 group was upregulated, while that in the KO-FAR591 group was downregulated (Fig. 4a, b). Then, we used methylprednisolone (MP; a GC commonly used for intramuscular injection) to establish the SONFH model in SD rats. Compared to that of the normal group without MP modeling, the expression of Fos, Bim, and Puma in BMECs of the femoral head in the NC-FAR591/MP group and the number of CD31^+^-TUNEL^+^ double-positive cells increased (CD31-labeled BMECs; Fig. 4c–j). Angiography showed that the microvessel volume and blood flow in the subchondral region of the femoral head decreased (Fig. 4k, l). Simultaneously, the expression levels of runt-related transcription factor 2 (Runx2) and OSX in the osteonecrotic area decreased (Fig. 5a–c), osteoblasts around the trabeculae disappeared, and there were numerous empty bone lacunae in the trabecular bone (Fig. 5d). Micro-CT showed that the trabecular arrangement in the osteonecrotic area was disordered, the bone resorption became thinner, and the BMD, BVF, Tb. N, and Tb. Th were decreased (Fig. 5f–j). TRAP staining showed an increase in the number of osteoclasts and bone resorptive activity (Fig. S5A–C). MRI showed abnormally high signals on T2-WI and low signals on T1-WI in the subchondral area of the femoral head, suggesting femoral head necrosis (Fig. 5e).

Compared to those of the NC–FAR591/MP group, the expression levels of Fos, Bim, and Puma in BMECs of the femoral head in the KO-FAR591/MP group and the number of CD31^+^-TUNEL^+^ double-positive cells decreased (CD31-labeled BMECs; Fig. 4c–j). Angiography showed that the microvessel volume and blood flow in the subchondral region of the femoral head increased (Fig. 4k, l). The expression levels of Runx2 and OSX increased (Fig. 5a, c), osteoblasts were observed around the trabeculae, and the empty bone lacunae in the trabeculae were obviously reduced (Fig. 5d). Micro-CT showed that the bone trabeculae were arranged regularly, and the BMD, BVF, Tb. N, and Tb. Th were increased (Fig. 5f–j). TRAP staining showed decreases in the osteoclast number and bone resorptive activity (Fig. S5A–C). MRI showed decreased abnormal signals on T1-WI and T2-WI in the subchondral area of the femoral head, indicating a reduction in osteonecrosis (Fig. 5e). These results showed that knockout of FAR591 blocked the apoptosis of BMECs induced by GCs, which reduced the damage of GCs to the femoral head microcirculation and inhibited the pathogenesis and progression of SONFH.

Compared to that of the NC-FAR591/MP group, the expression of Fos, Bim, and Puma in BMECs of the femoral head in the OE-FAR591/MP group and the number of CD31^+^-TUNEL^+^ double-positive cells increased (CD31-labeled BMECs; Fig. 4c–j). Angiography showed that the microvessel volume and blood flow in the subchondral region of the femoral head decreased (Fig. 4k, l). The expression levels of Runx2 and OSX decreased (Fig. 5a–c), the osteoblasts around the trabeculae disappeared, there were many empty bone lacunae in the trabecular bone, and the bone marrow tissue was extensively necrotic (Fig. 5d). Micro-CT showed that the subchondral trabeculae were widely absorbed and interrupted, and the BMD, BVF, Tb. N, and Tb. Th were significantly decreased (Fig. 5f–j). TRAP staining showed a significant increase in the number of osteoclasts and bone resorptive activity (Fig. S5A–C). MRI showed increased abnormal signals on T1-WI and T2-WI in the subchondral area of the femoral head, suggesting aggravated osteonecrosis (Fig. 5e). These results showed that overexpression of FAR591 promoted the apoptosis of BMECs induced by GCs, which aggravated the GC-induced damage to the microcirculation of the femoral head and promoted the pathogenesis and progression of SONFH.

### Fos, as the target gene of FAR591, promoted BMEC apoptosis by regulating Bim and Puma

**Fig. 6:**
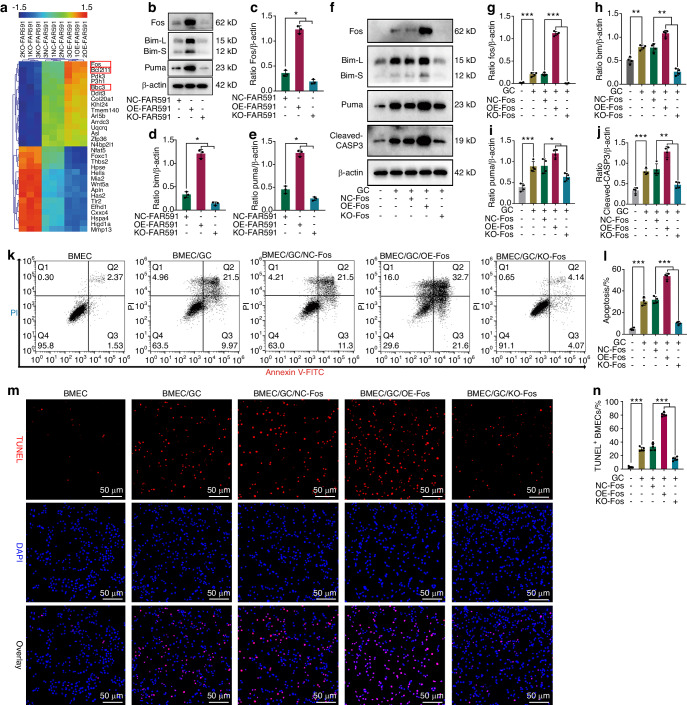
Fos, as a target gene of FAR591, can mediate GC-induced apoptosis of BMECs. In BMECs with overexpression and knockout of FAR591, the gene expression profile was detected by microarray: **a** mRNA cluster analysis (*n* = 3); the genes in the red box are Fos, Bcl2l11 (Bim), and Bbc3 (Puma). **b** The expression of Fos, Bim, and Puma in BMECs with overexpression or knockout of FAR591 was verified by western blots (*n* = 3). **c** Quantitative analysis of Fos expression shown in (**b**) (*n* = 3). **d** Quantitative analysis of Bim expression shown in (**b**) (*n* = 3). **e** Quantitative analysis of Puma expression shown in (**b**) (*n* = 3). After overexpression or knockout of Fos in BMECs, BMEC apoptosis was induced by GC (HC): **f** The expression of Fos, Bim, Puma, and cleaved CASP3 in BMECs with overexpression or knockout of Fos was verified by western blotting (*n* = 4). **g** Quantitative analysis of Fos expression shown in (**f**) (*n* = 4). **h** Quantitative analysis of Bim expression shown in (**f**) (*n* = 4). **i** Quantitative analysis of Puma expression shown in (**f**) (*n* = 4). **j** Quantitative analysis of cleaved CASP3 expression shown in (**f**) (*n* = 4). **k**, **l** Flow cytometry was used to detect BMEC apoptosis (*n* = 4). **m**, **n** TUNEL/DAPI was used to detect BMEC apoptosis (*n* = 5). In (**c–e**, **g–j**, **l**, **n**), data are presented as the means ± SDs; statistical significance was calculated by one-way ANOVA with Tukey’s *post hoc* tests; ^*^P < 0.05, ^**^P < 0.01, ^***^P < 0.001

To further study the mechanism of FAR591-mediated GC-induced apoptosis in BMECs, we used a microarray to detect gene expression profiles in BMECs with overexpression and knockout of FAR591 to screen downstream targets regulated by FAR591. The results showed that compared to that of the negative control group, the expression of Fos, Bim, and Puma was upregulated after overexpression of FAR591 and downregulated after knockout of the FAR591 gene (fold change >2, *P* < 0.05; Fig. 6a). The microarray data were verified by western blotting, and the results further confirmed the regulatory effect of FAR591 on the expression of Fos, Bim, and Puma (Fig. 6b–e). Studies have shown that Fos, acting as a stress transcription factor, can interact with transcriptional coactivators to regulate gene expression in mitochondrial apoptotic pathways such as Bim and Puma.^59,60^

Therefore, we further studied the role of Fos in GC-induced apoptosis of BMECs. To this end, the Fos gene was knocked out by CRISPR/Cas9 technology (KO-Fos), or the expression of Fos was upregulated by gene overexpression technology (OE-Fos), and then, BMEC apoptosis was induced by GCs. The results showed that compared to that of the BMECs without GC treatment, the expression of Fos, Bim, Puma, and cleaved CASP3 was upregulated after GC treatment (Fig. 6f–j), and the apoptotic rate of BMECs was significantly increased (*P* < 0.001; Fig. 6k–n). After Fos knockout, the expression of Bim, Puma, and cleaved CASP3 was downregulated, and the apoptotic rate of BMECs was significantly decreased compared to those of the negative control group (*P* < 0.05; Fig. 6f–n); in contrast, after upregulation of Fos expression, the expression of Bim, Puma, and cleaved CASP3 was upregulated, and the apoptotic rate of BMECs was significantly increased (*P* < 0.05; Fig. 6f–n). These results confirmed that Fos promoted GC-induced apoptosis of BMECs by regulating the expression of Bim and Puma.

### FAR591 mediated GC-induced apoptosis of BMECs by regulating the expression of Fos

**Fig. 7:**
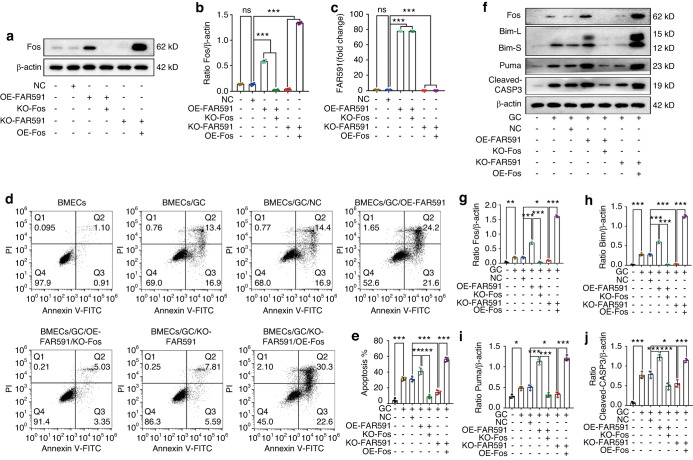
FAR591 mediated GC-induced apoptosis of BMECs by regulating Fos expression. The expression of FAR591 and Fos in BMECs was simultaneously interfered with by gene transfection technology or CRISPR/Cas9 technology: **a**, **b** Western blot detection of Fos (*n* = 4). **c** qPCR detection of FAR591 (*n* = 3). After successful intervention of FAR591 and Fos expression in BMECs, GCs (HC) were used to induce apoptosis of BMECs: **d**, **e** Flow cytometry was used to detect the apoptosis of BMECs (*n* = 4). **f** The expression levels of Fos, Bim, Puma, and cleaved CASP3 were detected by western blotting (*n* = 3). **g** Quantitative analysis of Fos expression shown in (**f**) (*n* = 3). **h** Quantitative analysis of Bim expression shown in (**f**) (*n* = 3). **i** Quantitative analysis of Puma expression shown in (**f**) (*n* = 3). **j** Quantitative analysis of cleaved CASP3 expression shown in (**f**) (*n* = 3). In (**b**, **c**, **e**, **g–j**), data are presented as the means ± SDs; statistical significance was calculated by one-way ANOVA with Tukey’s *post hoc* tests; ^*^P < 0.05, ^**^P < 0.01, ^***^P < 0.001

Fos promoted GC-induced apoptosis of BMECs by regulating the expression of Bim and Puma. As Fos is a downstream target regulated by FAR591, FAR591 mediates GC-induced apoptosis of BMECs, which may be achieved by regulating the expression of Fos. To verify this hypothesis, we designed blocking and rescue experiments and used gene transfection overexpression or CRISPR/Cas9 technology to simultaneously intervene in the expression of FAR591 and Fos genes in BMECs. Compared to that of the negative control group, Fos expression in the OE-FAR591 and KO-FAR591/OE-Fos groups was significantly upregulated, while that in the KO-FAR591 and OE-FAR591/KO-Fos groups was significantly downregulated (Fig. 7a, b). The expression of FAR591 was significantly upregulated in the OE-FAR591 and OE-FAR591/KO-Fos groups and significantly downregulated in the KO-FAR591 and KO-FAR591/OE-Fos groups (Fig. 7c).

After successful regulation of the expression of FAR591 and Fos in BMECs, GCs were used to induce BMEC apoptosis. Compared to that of the negative control group, the expression of Bim, Puma, and cleaved CASP3 was upregulated in the OE-FAR591 group (Fig. 7f–j), and the apoptotic rate of BMECs was increased (Fig. 7d, e). Compared to that of the OE-FAR591 group, the expression of Bim, Puma, and cleaved CASP3 was downregulated in the OE-FAR591/KO-Fos group (Fig. 7f–j), and the apoptotic rate of BMECs was decreased (Fig. 7d, e). These results showed that overexpression of FAR591 promoted GC-induced apoptosis of BMECs and that knocking out Fos on the basis of overexpression of FAR591 blocked the proapoptotic effect of FAR591.

Compared to that of the negative control group, the expression of Bim, Puma, and cleaved CASP3 in the KO-FAR591 group was downregulated (Fig. 7f–j), and the apoptotic rate of BMECs was decreased (Fig. 7d, e). Compared to that of the KO-FAR591 group, the expression of Bim, Puma, and cleaved CASP3 was upregulated in the KO-FAR591/OE-Fos group (Fig. 7f–j), and the apoptotic rate of BMECs was increased (Fig. 7d, e). These results showed that knockout of FAR591 inhibited GC-induced BMEC apoptosis, while overexpression of Fos on the basis of knockout of FAR591 reversed the results of knockout of FAR591 and promoted GC-induced BMEC apoptosis. In conclusion, the above results confirm that FAR591 mediates GC-induced apoptosis of BMECs by regulating the expression of Fos.

### FAR591 recruited TAF15 and Pol II in the Fos gene promoter to regulate Fos expression by transcriptional activation

**Fig. 8:**
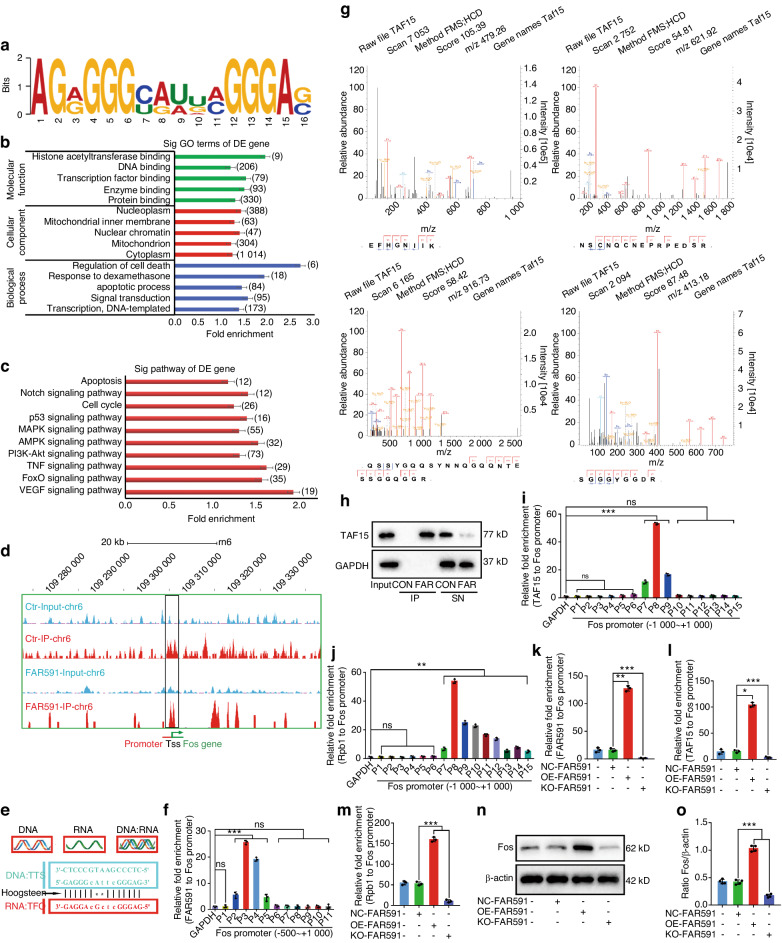
FAR591 recruited TAF15 and Pol II in the promoter region of the Fos gene to promote the expression of Fos by transcriptional activation. ChIRP-Seq detection of cis-acting elements interacting with FAR591 (*n* = 3): **a** Motif analysis results. **b** GO enrichment analysis. **c** KEGG enrichment analysis. **d** Visualization of FAR591 enrichment on chromosome 6. **e** Triplet diagram of FAR591 binding to the Fos gene promoter. **f** ChIRP-PCR was used to verify the binding site of FAR591 on the Fos gene promoter (*n* = 3). ChIRP-MS detection of trans-acting factors interacting with FAR591 (*n* = 3)**: g** MS identified four specific peptides in TAF15. **h** ChIRP-western blot analysis verified the interaction of FAR591 with TAF15 (*n* = 4). ChIP-PCR was used to detect the binding sites of TAF15 (**i**) and Rpb1 (**j**) on the Fos gene promoter (*n* = 3). After overexpression or knockout of FAR591 in BMECs: The enrichment levels of FAR591 (**k**), TAF15 (**l**), and Rpb1 (**m**) on the Fos gene promoter were detected by ChIRP/ChIP-PCR (*n* = 3). **n**, **o** The expression of Fos protein was detected by western blotting (*n* = 4). In (**f**, **i–m**, **o**), data are presented as the means ± SDs; statistical significance was calculated by one-way ANOVA with Tukey’s post hoc tests; ^*^P < 0.05, ^**^P < 0.01, ^***^P < 0.001. Abbreviations: chromatin isolation by RNA purification (ChIRP), high-throughput sequencing (Seq), triplex target site (TTS), triplex-forming oligonucleotide (TFO), mass spectrometry (MS), control (CON), FAR591 (FAR), immunoprecipitation (IP), supernatant (SN), RNA polymerase II subunit 1 (Rpb1)

FAR591 is mainly located in the nucleus (Fig. 2h). After overexpression or knockout of FAR591, the expression of Fos at the mRNA and protein levels changed significantly (Fig. S6). These results indicate that the regulatory effect of FAR591 on Fos may occur at the transcriptional level. Therefore, we designed ten specific oligonucleotide probes according to the FAR591 sequence (Table S1) and performed chromatin isolation by RNA purification (ChIRP) technology to isolate cis-acting elements and trans-acting factors interacting with FAR591. High-throughput sequencing (Seq) was used to screen cis-acting elements, and the results showed that 80523 differential enrichment peaks of FAR591 were detected in genomic DNA (Fig. S7A); 57.95% of these differential enrichment peaks were located between genes, 1.19% in exons, 25.51% in introns, 11.00% in upstream regions, and 4.35% in promoter regions (Fig. S7B); the enrichment peaks in the promoter region were mainly distributed near the transcription start site (TSS, TSS ± 5.0 kb; Fig. S7C). Motif analysis showed that the motif sequence combined with FAR591 is listed as AGRGGGYRWDMGGGAS (Fig. 8a). We further performed Gene Ontology (GO) and KEGG enrichment analyses on the genes where these enrichment peaks are located. GO enrichment analysis showed that the biological processes enriched by these genes mainly included cell death, apoptosis, response to dexamethasone, signal transduction, and DNA-dependent transcription processes (Fig. 8b). KEGG enrichment analysis showed that the signaling pathways enriched by these genes mainly included apoptosis- and proliferation-related signaling pathways (such as apoptosis, notch, p53, MAPK, AMPK, PI3K-Akt, TNF, FOXO, and VEGF; Fig. 8c). Both the FAR591 and Fos genes are located on chromosome 6, and the visual results of FAR591 enrichment on chromosome 6 showed that FAR591 was significantly enriched in the promoter region of the Fos gene (–500~ + 1 000; Fig. 8d). We designed 11 pairs of specific primers for the Fos gene promoter region (–500~+1 000; Table S2), verified the sequencing results by ChIRP-PCR, and determined that the binding site of FAR591 on the Fos gene promoter was located in the –245 to –51 region upstream of the Fos gene TSS (Fig. 8e, f).

Next, mass spectrometry (MS) was used to screen trans-acting factors. As a result, 26 proteins interacting with FAR591 were identified, which were related to transcriptional activation, including TATA-box binding protein associated factor 15 (TAF15; Fig. S7D–E). Four specific peptides were detected by MS in TAF15, with a sequence coverage of 21.2% (Fig. 8g). We used ChIRP-western blotting to verify the MS results and found that there was no positive band near 77 kDa for the protein isolated by the nonspecific antisense probe, while there was a positive band near 77 kDa for the protein isolated by the specific FAR591 probes (Fig. 8h). Simultaneously, we isolated the RNA interacting with TAF15 using an RNA binding protein immunoprecipitation technique and found that FAR591 was significantly enriched in the complex precipitated by the TAF15 antibody (Fig. 7F). These results confirmed the interaction between FAR591 and TAF15.

TAF15 is a stress transcriptional coactivator that activates gene expression by binding RNA polymerase II (Pol II).^61,62^ We designed 15 pairs of specific primers (Table S3) for the promoter region (–1 000–+1 000) of the Fos gene and identified the binding sites of TAF15 and Pol II on the Fos gene promoter by chromatin immunoprecipitation (ChIP). The results showed that the binding sites of TAF15 and Pol II on the Fos gene promoter were located near the TSS (–108–+80; Fig. 8i, j). Next, we studied whether FAR591 interfered with the binding of TAF15 and Pol II to the promoter of the Fos gene. The results showed that overexpression of FAR591 promoted the enrichment of TAF15 and Pol II in the promoter region of the Fos gene (Fig. 8k–m) and upregulated the expression of Fos (Fig. 8n, o). Knockout of FAR591 inhibited the enrichment of TAF15 and Pol II in the promoter region of the Fos gene (Fig. 8k–m) and downregulated the expression of Fos (Fig. 8n, o). These results showed that FAR591 recruited TAF15 and Pol II in the promoter region of the Fos gene, thereby activating its expression.

## Discussion

In this study, we found that FAR591 is a key regulatory molecule in GC-induced osteonecrosis of the femoral head. FAR591 participates in the pathogenesis and progression of SONFH by regulating the apoptosis of BMECs. Mechanistically, FAR591 responded to GR-mediated nuclear effects and recruited TAF15 and Pol II in the promoter region of the Fos gene to promote Fos gene expression through transcriptional activation. This process resulted in GC-induced BMEC apoptosis via activation of the Fos/mitochondrial apoptotic pathway, which eventually led to dysfunction of the femoral head microcirculation and the pathogenesis and progression of femoral head necrosis. To the best of our knowledge, this is the first report to elucidate the role and mechanism of FAR591 in the pathogenesis of SONFH. Therefore, FAR591 and its regulatory mechanism may become new targets for the early prevention and treatment of SONFH.

The coupling mechanism of bone microcirculation and osteogenesis is particularly important for maintaining bone homeostasis and initiating bone regeneration.^63^ The injury of the coupling mechanism is the core event of the pathogenesis and progression of SONFH, but the pre-event of coupling mechanism injury remains controversial.^43–48^ In this study, we first compared the sensitivity of BMECs and OBs to GCs in vitro. The results showed that a low concentration of GCs induced BMEC apoptosis and inhibited tubulogenesis, while a higher concentration of GCs was needed to induce OB apoptosis and inhibit osteogenesis. Second, we also studied the relationship between microcirculation injury and osteogenesis impairment in the pathogenesis and progression of SONFH in vivo. The results demonstrated that BMEC apoptosis and microcirculation dysfunction first appeared in GC-induced femoral head necrosis, followed by osteogenic damage, osteonecrosis, and increased bone resorption. Therefore, we believe that GC-induced BMEC apoptosis is the pre-event that destroys bone microcirculation and the osteogenic coupling mechanism and precipitates SONFH. Therefore, this study focused on BMECs rather than OBs when exploring the pathogenesis of SONFH.

GC-induced apoptosis is related to the abnormal expression of mitochondrial apoptotic pathway gene clusters, but the upstream regulatory mechanism of the abnormal expression of these gene clusters is still not fully understood.^23–28^ LncRNA is a type of noncoding RNA molecule with a transcript length of >200 nucleotides that plays an important role in regulating cell apoptosis and other life processes.^64–67^ At present, some studies have reported the role of lncRNAs in regulating the function of endothelial cells. For example, lncRNA CA7-4 promotes autophagy and apoptosis of vascular endothelial cells induced by high glucose,^68^ while lncRNA MANTIS (n342419) promotes the regeneration of vascular endothelial cells.^69^ In this study, a lncRNA/mRNA microarray was used to detect differentially expressed mRNAs in a cell model of GC-induced apoptosis of BMECs. The mRNA enriched in the apoptotic signal pathway was mainly that of the mitochondrial apoptotic pathway, which confirmed the above argument that “GC-induced apoptosis is related to the abnormal expression of the mitochondrial apoptotic pathway gene cluster.” Additionally, we identified a novel lncRNA in BMECs using the lncRNA/mRNA microarray, namely, FAR591, which has a significant coexpression relationship with the mitochondrial apoptotic pathway gene cluster and is significantly overexpressed in the process of GC-induced BMEC apoptosis and femoral head necrosis. Functional experiments confirmed that FAR591 is an upstream target responsible for regulating the abnormal expression of the mitochondrial apoptotic pathway gene cluster and plays a key role in promoting GC-induced BMEC apoptosis and femoral head necrosis.

The essence of lncRNAs is nucleic acids, and their participation in cellular activity is mainly achieved by regulating gene expression.^70–72^ In this study, we used a gene chip to detect the gene expression profile of BMECs with overexpressed or knocked out FAR591 to screen out whether Fos might be the downstream target of FAR591 and further confirmed the regulatory effect of FAR591 on Fos gene expression in BMECs. In the functional experiment with Fos, we confirmed that Fos mediated GC-induced BMEC apoptosis by regulating Bim and Puma expression. On this basis, we further designed blocking and rescue experiments; that is, on the basis of the upregulation of FAR591, we further knocked out Fos, which blocked the proapoptotic effect of FAR591. In contrast, after knocking out FAR591, we further upregulated Fos, which reversed the effects of FAR591 knockout. These results confirmed that the mechanism of FAR591-mediated GC-induced apoptosis of BMECs is mainly via the regulation of Fos expression.

Additionally, we studied the mechanism by which FAR591 regulates Fos gene expression. FAR591 is mainly located in the nucleus and regulates the mRNA and protein expression of Fos. Therefore, FAR591 may also regulate the expression of Fos at the transcriptional level. Studies have shown that lncRNAs mainly interact with cis-acting elements or trans-acting factors at the transcriptional level and then regulate gene expression by participating in histone modification, DNA methylation, transcriptional interference, and chromatin remodeling.^73–76^ In this study, specific oligonucleotide probes were designed according to the FAR591 sequence. Cis-acting elements or trans-acting factors interacting with FAR591 were isolated by ChIRP. Combined with the analysis of Seq and MS, we found that FAR591 could not only bind to the Fos gene promoter (–245 to –51) but also bind to the transcription factor TAF15. TAF15 is a stress transcription coactivator that recognizes and binds to the TATA box of the gene promoter, and its N-terminal low complexity (LC) domain directly binds to the carboxy-terminal domain of Pol II, thus recruiting Pol II to bind to the gene promoter, promoting the formation of the preinitiation complex (PIC) and activating gene transcription.^77,78^ We confirmed the binding site (–108 to +80) of TAF15 and Pol II on the Fos gene promoter using ChIP‒qPCR. Knockout of FAR591 blocked the enrichment of TAF15 and Pol II in the promoter region of the Fos gene and then downregulated the expression of Fos. Overexpression of FAR591 promoted the enrichment of TAF15 and Pol II in the promoter region of the Fos gene and then upregulated the expression of Fos. These results confirm that FAR591 regulates Fos gene expression mainly through the recruitment of TAF15 and Pol II in the promoter region of the Fos gene and then promotes Fos gene expression by transcriptional activation. Regarding the binding site of FAR591 on TAF15, studies have shown that the C-terminus of TAF15 contains an RNA recognition motif domain, which can bind RNA.^79,80^ However, the interaction site of TAF15 on FAR591 remains unclear. Our bioinformatics analysis shows that there may be two binding sites of TAF15 on FAR591, both of which are located at the 5′ end of FAR591 (Fig. S7G–I), although the results need to be further verified.

This study also has some limitations. First, we observed that the adult rat has a persistently open proximal femoral growth plate, which is different from the adult human with a closed growth plate. It is unclear whether this anatomical difference plays a role in SONFH. In this study, we did not observe significant changes in the growth plate at the early stage of osteonecrosis, although this issue requires further study. Second, the use of MRI to evaluate osteonecrosis is mainly based on the results of T1- and T2-weighted images, and quantitative analysis of the osteonecrotic area may be inaccurate. In contrast, enhanced MRI or necrosis-specific MRI sequences can provide more accurate quantitative results. Third, we used only male rats for the study, and it is unclear whether the results of this study apply to females. Since SONFH exists in both males and females, we should include both sexes in future research.

**Fig. 9:**
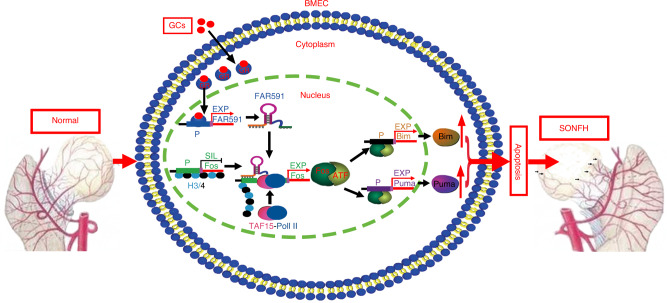
Schematic diagram of the mechanism of FAR591-mediated GC-induced femoral head necrosis. GCs interact with GR, and activated GR translocates into the nucleus to bind to the FAR591 gene promoter and activate FAR591 gene expression. FAR591 binds to the Fos gene promoter to form a stable RNA:DNA triplet structure. FAR591 recruits TAF15 and Pol II in the promoter region of the Fos gene, which promotes preinitiation complex formation and Fos gene expression by transcriptional activation. Fos activates the mitochondrial apoptosis pathway by regulating Bim and Puma and then mediates GC-induced BMEC apoptosis, which ultimately leads to bone microcirculation dysfunction and the pathogenesis and progression of SONFH. Abbreviations: expression (EXP), silent (SIL), promoter (P)

In conclusion, we determined that GC-induced apoptosis of BMECs is a pre-event in the pathogenesis and progression of SONFH. Simultaneously, we identified a novel lncRNA in BMECs, FAR591, which was significantly overexpressed in the process of GC-induced BMEC apoptosis and femoral head necrosis. FAR591 also mediates GC-induced BMEC apoptosis, thus aggravating GC-induced damage to the femoral head microcirculation and promoting the pathogenesis and progression of SONFH. Mechanistically, FAR591 responded to GR-mediated nuclear effects and was induced to be highly expressed by GCs in BMECs. Then, FAR591 binds to the promoter of the Fos gene to form a stable RNA:DNA triplet structure and promotes the formation of PIC in the promoter of the Fos gene by recruiting TAF15 and Pol II, which activates the expression of the Fos gene. Fos promotes the gene expression of mitochondrial apoptotic pathways such as Bim and Puma, which mediates GC-induced BMEC apoptosis (Fig. 9). Although the interaction site between FAR591 and TAF15 needs further verification, our current in vitro and in vivo experimental results have confirmed that the FAR591/Fos signaling axis plays a vital role in the pathogenesis and progression of SONFH. This study adds new evidence to reveal the pathogenesis of SONFH and opens up new targets for the early prevention and treatment of SONFH.

## Materials and methods

### Animal experiments

All SD rats were provided by the experimental animal center of Guizhou Medical University, and the animal experiments were completed in the center. The certificate number of the experimental facility is SYXK (qian) 2018-0001. All procedures with animals were performed in accordance with the guidelines on the use and care of laboratory animals for biomedical research published by the National Institutes of Health (No. 85-23, revised 1996), and the experimental protocol was reviewed and approved by the ethical committees of Guizhou Medical University (No. 2101122).

### Culture and identification of BMECs

The cancellous bone of the femoral head of male SD rats weighing 90 ± 10 g was broken into fragments, and the fat and connective tissue in the cancellous bone were cleaned with phosphate buffered saline (PBS; Gibco, USA). The bone tissue was digested with 0.2% type I collagenase (Sigma‒Aldrich, Germany) and 0.1% trypsin (Gibco) at 37 °C. After the bone remnants were filtered, the cell suspension was centrifuged for 5 min at 2 000 r·min^−1^, and the cell precipitate was divided into two layers. The cells in the lower layer were inoculated with endothelial cell medium (ECM; ScienCell, USA) in a 2% gelatin-coated culture flask (Solarbio, China) and cultured at 37 °C and 5% CO_2_. The expression of von Willebrand factor (vWF) was identified by immunofluorescence staining (antibodies were purchased from Santa Cruz, USA), and cluster of differentiation 31 (CD31) was identified by flow cytometry (antibodies were purchased from BD Biosciences, USA). Third-generation BMECs were used for in vitro experiments.

### GC-induced apoptosis model of BMECs

Third-generation BMECs were treated with different concentrations of hydrocortisone (HC; 0 mg·mL^−1^, 0.1 mg·mL^−1^, 0.2 mg·mL^−1^, 0.3 mg·mL^−1^, 0.4 mg·mL^−1^; Kingyork, China) for 24 h. The apoptosis of BMECs was detected by terminal deoxynucleotidyl transferase (TdT)-mediated dUTP nick-end labeling (TUNEL)/4′,6-diamidino-2-phenylindole (DAPI) double staining (Beyotime, China) and Annexin V-fluorescein isothiocyanate (FITC)/propidium iodide (PI) double staining (BD Biosciences).

### LncRNA/mRNA microarray and bioinformatics analyses

Total RNA of BMECs was extracted by TRIzol (Invitrogen, USA) and quantified by a NanoDrop-2000 (ND2000, Nanodrop, USA). The RNA quality was assessed by formaldehyde agarose gel (Biowest, Spain) electrophoresis. First, ribosomal RNA was removed, and cDNA was synthesized. The synthesized cDNA was labeled and hybridized to an SD rat lncRNA/mRNA expression microarray v3.0 (8 × 60 K, Arraystar, USA). Microarrays were scanned with a chip scanner (G2565BA, Agilent, USA), and raw data were extracted by Agilent Feature Extraction software (12.0, Agilent). The differentially expressed genes were identified by a random variance model. A paired t test was used to calculate *P* values. The thresholds for up- or downregulated genes were fold change (FC) > 2.0 and *P* < 0.05. The expression patterns of lncRNAs and mRNAs were analyzed by hierarchical clustering using MultiExperiment Viewer software (4.9.0, Institute of Genomic Research, USA). Cytoscape software (3.8.0, National Institute of General Medical Sciences [NIGMS], USA) was used to analyze the coexpression network of lncRNAs and mRNAs (Pearson correlation coefficient >0.99, *P* < 0.01). The differentially expressed mRNAs were enriched by KEGG and GSEA (FC > 2, *P* < 0.05).

### Real-time quantitative PCR

The total RNA of BMECs was extracted by TRIzol. Then, M-MuLV RT Master Mix (Sangon Biotech, China) was used to reverse transcribe cDNA on a PCR amplification instrument (C1000, Bio-Rad, USA). The cDNA synthesis system is shown in Table S4. Finally, gene-specific primers (Sangon Biotech) and 2xSG Fast RT‒PCR Master Mix (Sangon Biotech) were used for PCR amplification on a real-time quantitative PCR iCycler iQ instrument (Bio-Rad). The qPCR system is shown in Table S5, and the gene-specific primers are shown in Table S6. We calculated the fold change in RNA expression compared to that of the control using the ΔΔCt method.

### Rapid amplification of the cDNA end

Total RNA was extracted by TRIzol, 28S and 18S were detected by formaldehyde agarose gel electrophoresis, and the A260/A280 ratio was determined by a NanoDrop-2000. For cloning of the partial sequence of FAR591, the partial sequence of FAR591 was amplified by a specific primer (Sangon Biotech) with cDNA of FAR591 as a template. The primer sequence is shown in Table S7, and the amplification system is shown in Table S8. Then, agarose gel electrophoresis was performed, and the gel was cut and recovered. The products were ligated into the pGM-T vector (Sangon Biotech) and transformed into highly efficient chemically competent DH5α cells. Finally, sequencing was performed to obtain sequence information. For the 5′ and 3′ RACE experiments of FAR591, 5′ and 3′ RACE primers were designed using the known sequences of FAR591 described above. The primer sequences are shown in Tables S9–10. PCR amplification was performed using RACE primers (Sangon Biotech) and the GeneRacer^TM^ Kit (Invitrogen). The amplification systems are shown in Tables S11–14. Then, the amplified product was recovered by agarose gel electrophoresis and gel cutting. The products were ligated to the pGM-T vector and transformed into highly efficient chemically competent DH5α cells. Finally, the sequence information of the 5’ and 3’ ends was obtained by sequencing. The sequencing results were spliced and compared to the known sequences of the genome.

### RNA fluorescence in situ hybridization

According to the instructions of the manufacturer of the RNA-FISH Kit (Vysis, USA), after the cells were fixed with 4% paraformaldehyde (Solarbio) and permeabilized with 0.5% Triton X-100 (Solarbio), the prehybridization solution was added and blocked at 37 °C for 30 min. FISH Probe Mix from lncRNA or reference (20 μmol·L^−1^) was added to the hybridization solution and hybridized overnight at 37 °C in the dark. The cells were washed with saline sodium citrate (SSC; Solarbio) and incubated with DAPI (10 μg·mL^−1^; Solarbio) for 8 min in the dark. The cells were washed with PBS, sealed with antifluorescence quenching agent (Southern Biotech, USA), and observed and photographed with a laser confocal microscope in the dark. The probe sequence is shown in Table S1.

### Transfection of lentivirus and screening of cloned cell lines

FAR591 gene overexpression lentivirus (GV367, 1E + 9 T U·mL^−1^), Fos gene overexpression lentivirus (GV367, 8E + 8 T U·mL^−1^), FAR591 gene knockout lentivirus (CV279, 5.5E + 8 T U·mL^−1^), Fos gene knockout lentivirus (CV279, 6E + 8 T U·mL^−1^), negative control lentivirus, and Hitrans P were purchased from GeneChem in Shanghai, China. Briefly, according to the optimal multiplicity of infection (MOI = 80) shown in the pre-experiment, lentivirus and Hitrans P were added to infect BMECs for 14 h. After 5 days, fluorescence was observed by inverted fluorescence microscope (DM1LLED, Zeiss), and the transfection efficiency was calculated. Stable clonal cell lines were obtained by continuous selection with puromycin (2 μg·mL^−1^; Beyotime). The efficiency of gene knockout was detected by the nonmismatch enzyme method. Genomic DNA was amplified by nested PCR, and positive bands between 500 bp and 1 000 bp were detected by agarose gel electrophoresis. The nested PCR primers are shown in Table S15, and the nested PCR system is shown in Tables S16 and S17. Finally, qPCR was used to verify the expression level of RNA. The FAR591-specific primers are shown in Table S6, and the Fos-specific primers are shown in Table S18. The primers and PCR-related reagents were purchased from Sangon Biotech (Shanghai, China).

### Western blot

The total protein of cells was extracted by radioimmunoprecipitation buffer (RIPA; Solarbio), and the protein was quantified by quinolinolic acid (Solarbio). SDS‒PAGE gels (Solarbio) were prepared, and the preparation systems of 12% separation gel and 5% concentrated gel are shown in Tables S19 and S20. An equal amount of denatured protein was added for electrophoresis. After electrophoresis, the gel was cut according to the manufacturer’s instructions (Thermo Scientific, USA), and the protein was transferred to a polyvinylidene fluoride membrane (PVDF; Merck, Germany). Subsequently, the blots were blocked with 5% bovine serum albumin (BSA; Solarbio) for 2 h, c-Fos (9F6) rabbit mAb (1:1 000; 2250; Cell Signaling Technology, USA), c-Jun (60A8) rabbit mAb (1:1 000; 9165; Cell Signaling Technology), cleaved Caspase-3 (Asp175) (5A1E) rabbit mAb (1:1 000; 9664; Cell Signaling Technology), Bim (C34C5) rabbit mAb (1:1 000; 2933; Cell Signaling Technology), Puma (E2P7G) rabbit mAb (1:1 000; 98672; Cell Signaling Technology), TAF15 (D8V6Q) rabbit mAb (1:1 000; 28409; Cell Signaling Technology), β-Actin (13E5) rabbit mAb (1:1 000; 4970; Cell Signaling Technology), and GAPDH (14C10) rabbit mAb (1:1 000; 2118; Cell Signaling Technology) were used in the primary antibody reaction and incubated overnight at 4 °C. After the PVDF membrane was washed with TBST buffer (Solarbio), HRP-conjugated mouse anti-rabbit IgG (1:2 500; 5127; Cell Signaling Technology) was used for the secondary antibody reaction, and the solution was incubated at 25 °C for 2 h. Subsequently, the PVDF membrane was washed with TBST buffer, and enhanced chemiluminescence (ECL; Millipore, Germany) was evenly coated on the PVDF membrane. The images were exposed and collected using a gel imaging system (GenoSens1880, Clinx Science Instruments, Ltd., China), and protein quantification was performed using ImageJ software (1.4.3.67, National Institutes of Health [NIH], USA).

### Chromatin isolation by RNA purification

According to the instructions of the manufacturer of the ChIRP kit (Merck), after cells were crosslinked with 37% formaldehyde (Sigma‒Aldrich) and terminated with glycine (125 mmol·L^−1^; Solarbio), cell lysate was added, and chromatin was broken by ultrasound before collecting the supernatant. First, biotin-labeled probes (specific FAR591 antisense as the experimental group, nonspecific RNA antisense as the control group, and FAR591 probe sequence as shown in Table S1) were combined with magnetic beads and then hybridized with chromatin overnight at 37 °C. The magnetic bead mixture was washed, and 1/20 of the sample was reserved for qPCR analysis. The magnetic bead mixture was eluted, and the supernatant was collected for high-throughput sequencing and mass spectrometry analysis.

### High-throughput sequencing

Proteinase K (Sigma‒Aldrich) was added to the ChIRP-separated chromatin, and DNA was extracted and purified using Ezup centrifugal columns (Sangon Biotech). The TruSeq Nano DNA sample preparation kit (Illumina, USA) was used for terminal repair and ligation of DNA samples. AMPure XP beads (Illumina) were used to select DNA fragments of ~200–1 500 bp, and the library size was determined by an Agilent 2100 Bioanalyzer (2100, Agilent, USA). According to the instructions of the NovaSeq 6000 S4 kit (Illumina), the bridged PCR was clustered on an Illumina cBot (Illumina) and sequenced on an Illumina NovaSeq 6000 (NovaSeq 6000, Illumina). Then, image analysis, base interpretation, and quality filtering were performed. Finally, alignment with the rat genome (UCSC RN6) was performed for peak detection and peak annotation.

### Mass spectrometry

The nuclease benzonase (20 U; Sigma‒Aldrich) was added to the ChIRP-separated chromatin, and the protein was extracted and purified. The protein was enzymatically hydrolyzed into specific peptides by trypsin (Promega, USA) and desalted by a balanced C18 desalination column (Sigma-Aldrich), and the peptides were used for LC‒MS/MS detection. Briefly, the peptides were separated by nano-UPLC (Thermo Scientific) and then analyzed by Q-Exactive Mass Spectrometry (Thermo Scientific). Raw data were library-searched and quantified using MaxQuant (1.6.1.0, Max Planck Institute of Biochemistry, Germany). The specific enzyme was trypsin/P, the maximum number of missing cuts was 3, the variable modifications included oxidation (M) and acetylation (protein N-term), and the fixed modification was carbamidomethyl (C). The quantitative method was MS1, the peptide used for quantification was “Unique,” and the variably modified peptide was not used for quantification. iBAQ nonstandard quantification was conducted in parallel, and the false discovery rate (FDR) of peptides and proteins was controlled at 0.01. Finally, the enriched proteins were analyzed by GO, KEGG, and protein‒protein interaction analyses.

### Chromatin immunoprecipitation

According to the instructions of the manufacturer of the ChIP Kit (Abcam, UK), the cells were crosslinked with 37% formaldehyde (Sigma‒Aldrich) and terminated with glycine (125 mmol·L^−1^; Solarbio). Next, cell lysis buffer was added, the chromatin was broken by ultrasound, and then, the supernatant was collected. The effect of ultrasonic crushing was detected by agarose gel electrophoresis. Protein A-salmon sperm DNA agar was used to remove nonspecific binding molecules with protein A agarose. The supernatant was collected, and 20 μL was taken as input DNA. Then, TAF15 (D8V6Q) rabbit mAb (1:50; 28409; Cell Signaling Technology), Rpb1 NTD (D8L4Y) rabbit mAb (1:50; 14958; Cell Signaling Technology), and rabbit (DA1E) mAb IgG (1:50; 3900; Cell Signaling Technology) were added and incubated overnight at 4 °C. Protein A-salmon sperm DNA agar was added and incubated at 4 °C to bind the antibody-transcription factor-DNA complex. The complexes were washed and eluted sequentially, and the supernatant was collected. Proteinase K (Sigma‒Aldrich) was added to the supernatant and input DNA, after which the DNA was recovered using Ezup centrifugal columns (Sangon Biotech), and Fos promoter-specific primers (Sangon Biotech) were used for PCR amplification to analyze the enrichment of transcription factors on the Fos promoter. The primer sequence of the Fos promoter is shown in Table S3.

### Adeno-associated virus infection in vivo

FAR591-overexpressing adeno-associated virus (AAV9, 6.5E + 12 v.g per mL), FAR591 gene knockout adeno-associated virus (4E + 12 v.g per mL), and negative control adeno-associated virus were purchased from GeneChem in Shanghai, China. Male SD rats weighing 450 ± 10 g were used for the experiments, and we used a random number table to divide the SD rats into the following four groups: NS, NC-FAR591, OE-FAR591, and KO-FAR591, with 30 SD rats in each group. Briefly, 10% chloral hydrate (3.5 mL·kg^−1^; League, China) was used for abdominal anesthesia. After disinfection, the skin, muscle, and peritoneum were incised in the mid-abdomen, and the abdominal aorta was isolated and punctured. Next, the needle tip was placed approximately 2 cm above the bifurcation of the left and right common iliac arteries, and the virus solution was slowly injected at a uniform speed. Each rat was injected with a virus dose of 3 × 10^11 ^v g, with a volume of 100–200 μL. The abdominal cavity was closed, and intake of food was forbidden for 6 h. After 2 weeks, the infection was repeated once. Four weeks after the second infection, the expression of FAR591 in BMECs was detected by RNAscope. Following overexpression or knockout of FAR591, the early SONFH model was established by MP.

### RNAscope technology

At the 4th week after adeno-associated virus infection in vivo, the femoral head was stained with anti-CD31 antibody [RM1006] (1:100; ab281583; Abcam) to label BMECs using immunofluorescence. Then, the specific double Z probe designed by Advanced Cell Diagnostics (Newark, CA, USA) was hybridized with FAR591. According to the manufacturer’s instructions, the RNAscope Multiplex Fluorescent Reagent Kit (Advanced Cell Diagnostics) was used for signal amplification and detection. The images were observed by a BX53 microscope (Olympus, Japan), and the images were taken by a cellSens standard electronic system (Olympus, Tokyo, Japan). The ratio of CD31^+^ cells coexpressing FAR591 to total CD31^+^ cells was calculated in each section.

### Early SONFH model

During adeno-associated virus infection in vivo, approximately 1/3 of SD rats in each group died due to anesthesia and surgical trauma, and ~20 SD rats remained in each group. The remaining SD rats were used to establish the SONFH model, and 20 healthy adult male SD rats were randomly selected as healthy controls. The rats were divided into the following five groups: normal, NS/GC, NC-FAR591/GC, OE-FAR591/GC, and KO-FAR591/GC, with 20 SD rats in each group. Briefly, the buttocks of the rats were dehaired and sterilized with alcohol before injection of MP (60 mg·kg^−1^; Pfizer, USA) into the gluteal muscle once a day for 10 days. The animals were weighed before each injection. At the 4th week after MP treatment, the blood supply and osteonecrosis of the femoral head were detected.

### MRI

The SD rats (16 per group) were anesthetized by intraperitoneal injection of 10% chloral hydrate (3.5 mL·kg^−1^; Leagene). A Bruker 7.0 T magnetic resonance instrument (the selected imaging coil was the cage RF signal transmitting coil with an inner diameter of 72 mm, and the array surface echo signal receiving coil with an inner diameter of 56 mm; PharmaScan70/16, Bruker, Germany) was used for scanning with the following parameters: repetition time, 2 200 ms; echo time, 35 ms; scan time, 2 520 s; field of view, 64 mm × 64 mm; matrix size 256 × 256; slice thickness, 1 mm. T1- and T2-weighted images were collected separately. Magnetic resonance images were processed using RadiAnt DICOM Viewer software (2020.2.3, Medixant, Poland), and the areas with high signal intensity on T2-WI and low signal intensity on T1-WI in the subchondral area were selected as the regions of interest.

### Angiography

Systemic blood was heparinized by intraperitoneal injection of heparin sodium (1 U·g^−1^; Sigma‒Aldrich). Anesthesia was performed by intraperitoneal injection of 10% chloral hydrate (3.5 mL·kg^−1^; Leagene). The skin, muscle, and peritoneum were incised at the abdominal midline to expose the abdominal aorta and inferior vena cava. The proximal end of the abdominal aorta was ligated above the left renal artery, and an 18 G indwelling needle was used to puncture the abdominal aorta toward the distal end. The inferior vena cava was ligated proximally and severed distally. The abdominal aorta was perfused with heparinized saline (50 U·mL^−1^) and 10% neutral formalin (Solarbio) in turn and then perfused at a constant rate (3 mL per minute) with Microfil contrast agent (diluent: 122 yellow: curing agent = 4:5:0.5; Flow Tech, Inc., USA) until the effluent from the inferior vena cava turned yellow. The corpses of the rats were placed overnight at 4 °C. On the second day, the femoral heads were removed and placed in 10% EDTA (Solarbio) and then decalcified at 37 °C for 2 months. The microvessels of the femoral head were reconstructed using micro-CT.

### Micro-CT

For micro-CT, the femoral head was fixed with 4% paraformaldehyde (Solarbio) for 48 h. A Bruker high-resolution micro-CT system (SkyScan1276, Bruker) was used to scan the femoral head under the following uniform conditions: current, 200 μA; voltage, 85 kV; resolution, 6.5 μm; exposure time, 384 ms; and scanning angle, 180°. The original image was obtained, and N-Recon software (V1.7.4.2, Bruker) was used to perform 3D image reconstruction under the following settings: smoothing, 3; beam-hardening, 5; and ring artifacts, 30%. The femoral head necrosis area was taken as the region of interest (ROI). The Tb. N, Tb. Th, BVF, and BMD of the ROI were analyzed using CT Analyzer software (1.18.8.0, Bruker).

### H&E staining

The bone tissue was decalcified with 10% EDTA (Solarbio), dehydrated with an alcohol gradient (Sinopharm, China), made transparent using xylene (Sinopharm), and embedded in paraffin (Sinopharm). The bone tissue was sliced with a Leica pathological slicer (RM2016, Leica, Germany) and baked at 60 °C for 3 h. The slices were dewaxed in xylene and gradient alcohol in turn. The dewaxed sections were stained with Mayer’s hematoxylin and 1% water-soluble eosin solution (Solarbio). The slices were dehydrated in graded alcohol, rendered transparent in xylene, and finally sealed with neutral gum (Sinopharm). Images were collected by using a BX53 microscope (Olympus, Japan).

### TUNEL staining

According to the manufacturer’s instructions for the TUNEL staining kit (Vazyme, China), the bone tissue was decalcified, dehydrated, rendered transparent, waxed, embedded, sliced, baked, and dewaxed (the procedure was the same as that outlined for H&E staining). Next, 100 μL of proteinase K solution (20 μg·mL^−1^, Sigma‒Aldrich) was added to each section and incubated at 37 °C for 20 min. Following incubation, TdT incubation buffer was added to the sections and allowed to incubate for 60 min at 37 °C in the dark. The preparation of the TdT incubation buffer is shown in Table S21. Following incubation, the sections were incubated with DAPI (20 μg·mL^−1^; Solarbio) in the dark for 5 min. Following washing, the sections were sealed with an antifluorescence quencher, and images were collected by biomicroscopy.

### TRAP staining

According to the manufacturer’s instructions for the TRAP staining kit (Wako, Japan), the bone tissue was decalcified, dehydrated, rendered transparent, waxed, embedded, sliced, baked, and dewaxed (the procedure was the same as that outlined for H&E staining). Next, TRAP staining solution was added, the slices were incubated at 37 °C for 30 min, and AMPD-HCL (pH = 9.4) was added and incubated for another 10 min. The nuclei were restained with methyl green, washed with double distilled water, and dehydrated with anhydrous ethanol. The slices were sealed after drying, and the images were collected by biomicroscopy.

### Immunofluorescence

Bone tissue was decalcified, dehydrated, rendered transparent, waxed, embedded, sliced, baked, and dewaxed (the procedure was the same as that outlined for H&E staining). Antigen retrieval was performed using the microwave method, and bone tissue antigen retrieval solution was purchased from BioShun (Shanghai, China). Bone tissues were blocked with goat serum (Solarbio) at 25 °C for 30 min. Anti-CD31 antibody [RM1006] (1:100; ab281583; Abcam), anti-Puma antibody (1:50, AF1204, Beyotime), anti-c-Fos antibody [1D10] (1:100, ZRB457, Sigma‒Aldrich), anti-Bim antibody [Y36] (1:100; ab32158; Abcam), anti-RUNX2 antibody [EPR22858-106] (1:1 000; ab236639; Abcam), and anti-Osterix antibody [EPR21034] (1:500; ab209484; Abcam) were used for the primary antibody reaction (incubated overnight at 4 °C). Goat anti-rabbit IgG (1:1 000; Alexa Fluor® 488, ab150077; Alexa Fluor® 647, ab150079; Abcam) was used for the secondary antibody reaction (incubated at 37 °C for 2 h). DAPI was used to label the nucleus. Finally, the slides were mounted with antifluorescence quencher, and the images were collected using a BX53 microscope (Olympus).

### Statistical analysis

SPSS 22.0 statistical software was used to analyze data, and GraphPad Prism 6.0 software was used to draw statistical graphs. For quantitative data, the Kolmogorov–Smirnov test was used to test the normality of the data, and analysis of variance was used to test the homogeneity of variance between groups. The data with a normal distribution and homogeneous variance are described as the mean ± standard deviation (SD). Two-tailed unpaired Student’s t test was used for analyses involving only two groups for comparison, and one-way ANOVA with Tukey’s *post hoc* test was used for analyses involving more than two groups. The data that were not normally distributed are described by the median (P25, P75). The Kruskal–Wallis rank-sum test with the DSCF method was used for comparisons between groups. *P* values < 0.05 indicate that the difference is statistically significant. For qualitative data, the data are described by the number of cases (percentage), and the comparison between groups was performed by Pearson’s χ2 test or Fisher’s exact test. If the difference between groups was statistically significant, the Bonferroni method was further used for pairwise comparison, and the *P* value was corrected. *P* < 0.05/times of pairwise comparison indicates that the difference is statistically significant.

### Supplementary information

Supplementary Materials

## Data Availability

Additional data or reagents are available from the corresponding author upon reasonable request.
