# Author Correction: FAR591 promotes the pathogenesis and progression of SONFH by regulating Fos expression to mediate the apoptosis of bone microvascular endothelial cells

**DOI:** 10.1038/s41413-024-00339-3

**Published:** 2024-05-20

**Authors:** Fei Zhang, Lei Wei, Lei Wang, Tao Wang, Zhihong Xie, Hong Luo, Fanchao Li, Jian Zhang, Wentao Dong, Gang Liu, Qinglin Kang, Xuesong Zhu, Wuxun Peng

**Affiliations:** 1 Department of Emergency Orthopedics, The Affiliated Hospital of Guizhou Medical University, Guiyang, Guizhou 550004 China; 2 School of Clinical Medicine, Guizhou Medical University, Guiyang, Guizhou 550004 China; 3 Department of Orthopedics, Rhode Island Hospital, Brown University, Providence, RI 02903 USA; 4 Department of Orthopedics, Shanghai Jiao Tong University Affiliated Sixth People’s Hospital, Shanghai, 200233 China; 5 Department of Orthopedics, The First Affiliated Hospital of Soochow University, Suzhou, Jiangsu 215000 China

**Keywords:** Bone, Pathogenesis

Correction to: *Bone Res* 10.1038/s41413-023-00259-8, published online 22 May 2023

Following publication of the original article^1^, the authors regrettably identified inadvertent mistakes in Fig. 6, Fig. 7, Fig. S1, and Fig. S2 and typesetting mistakes in Fig. 3, Fig. 4, Fig. 6 and Fig. 8. In Fig. 7d, the last image was not labelled with Q1, Q2, Q3, Q4, and their presented percentages. In Figs. 6m and 6n, Figs. S1A, S1B, S1I and S1D, and Figs. S2A and S2E, these images were used inaccurately. Although these modifications do not affect the conclusion in the article, all of the authors agree to rectify these inadvertent mistakes by providing corrected figures, to guarantee the accuracy of this research. The authors sincerely regret and apologize for any inconvenience caused.

The original figure 6m and 6n were:
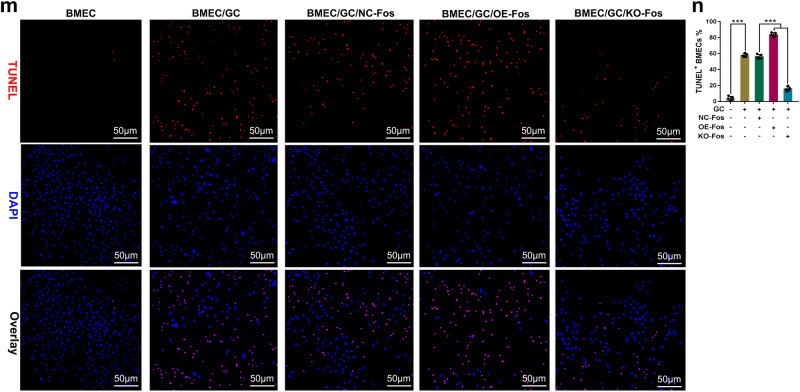


The original figure 7d was:
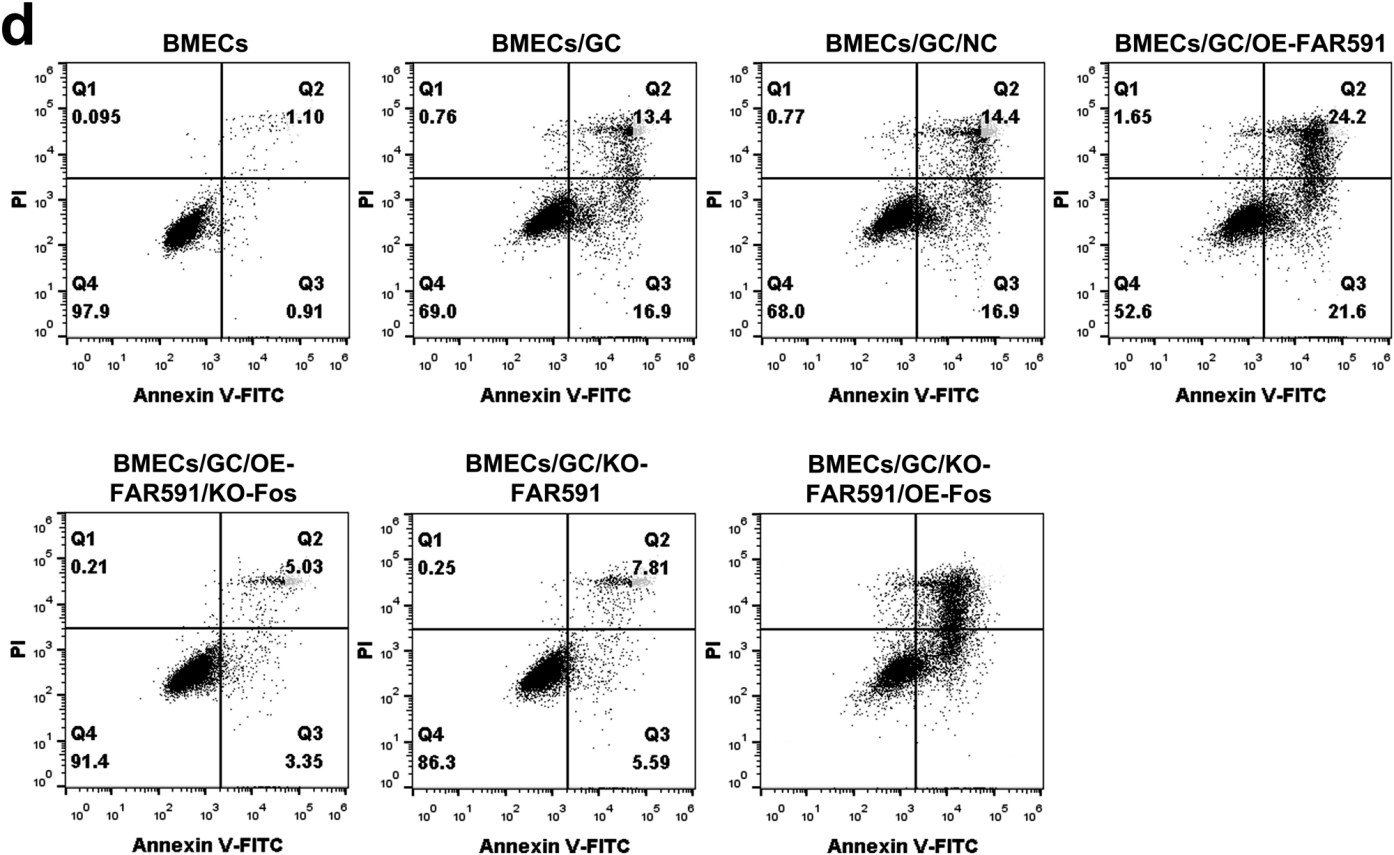


The original figure S1A, S1B, S1I and S1D were:
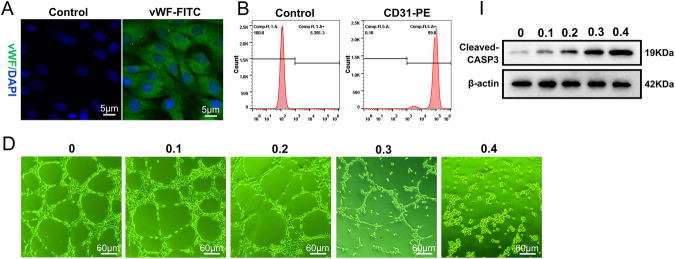


The original figure S2A and S2E were:
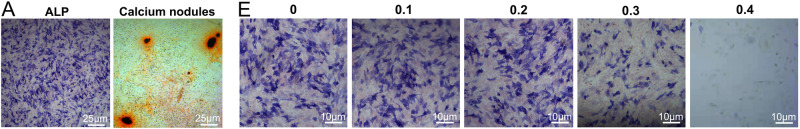


The correct figure 6m and 6n should read:
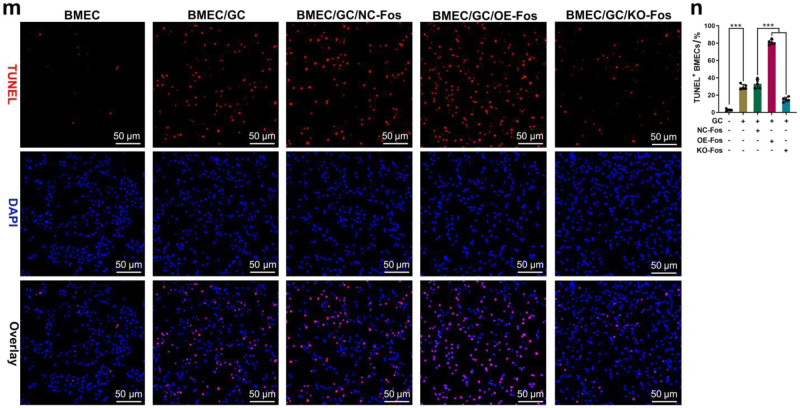


The correct figure 7d should read:
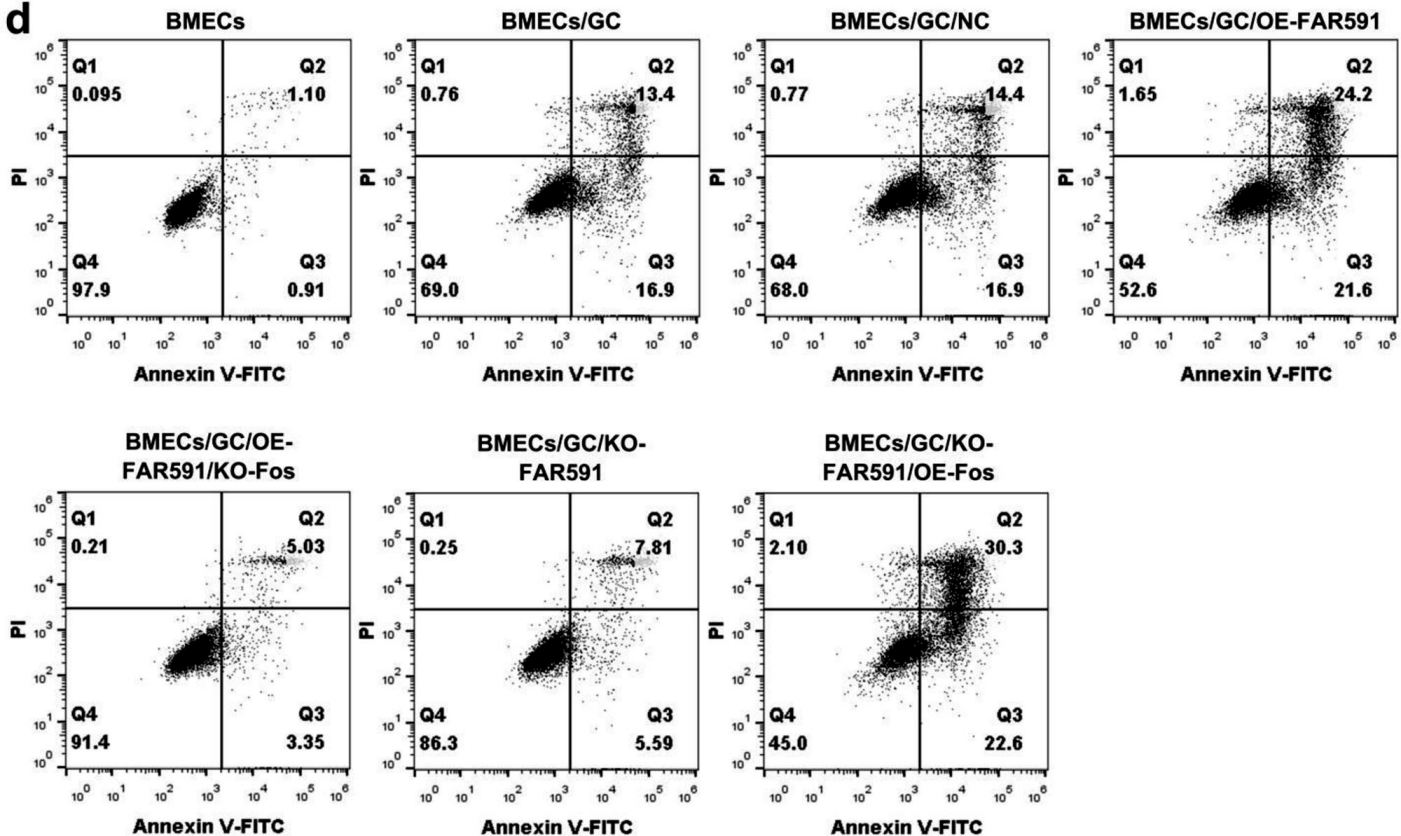


The correct figure S1A, S1B, S1I and S1D should read:
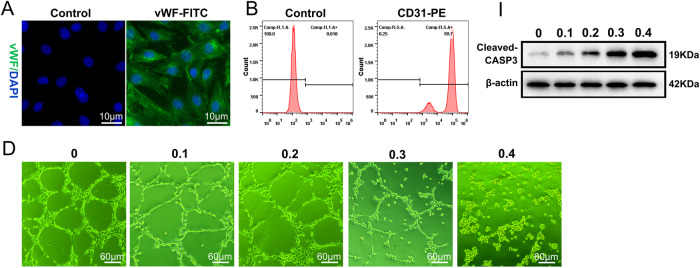


The correct figure S2A and S2E should read:
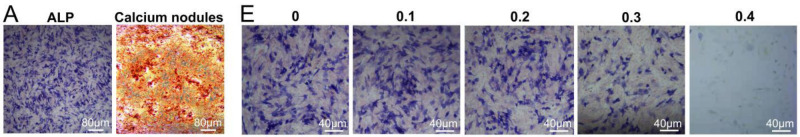


Due to a typesetting mistake, in Fig. 3e, 4d, 6d and 8m, some marked asterisks (*) are missing.

The original figure 3e was:
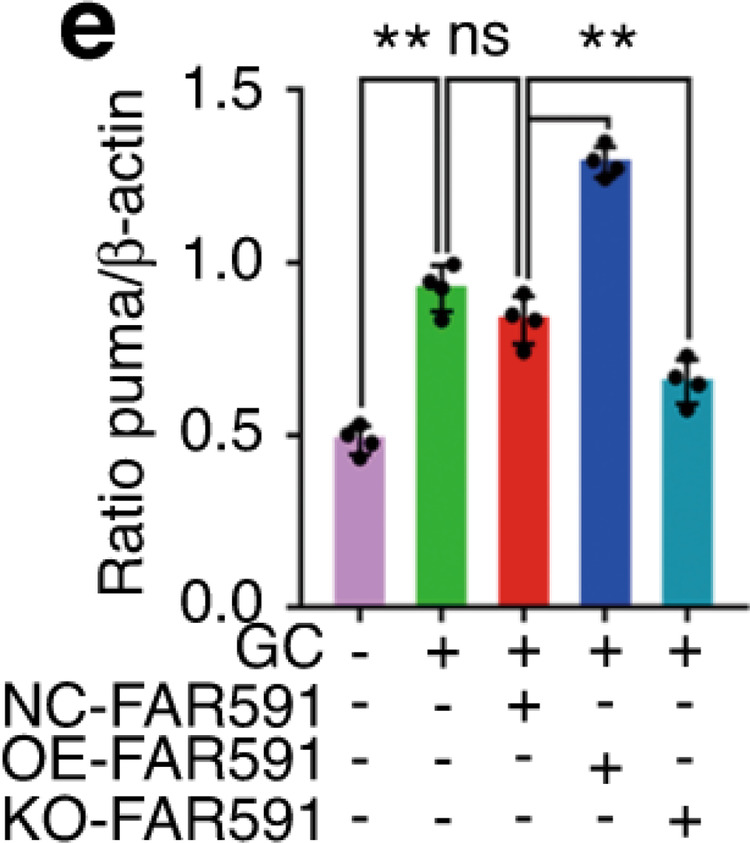


The correct figure 3e should read:
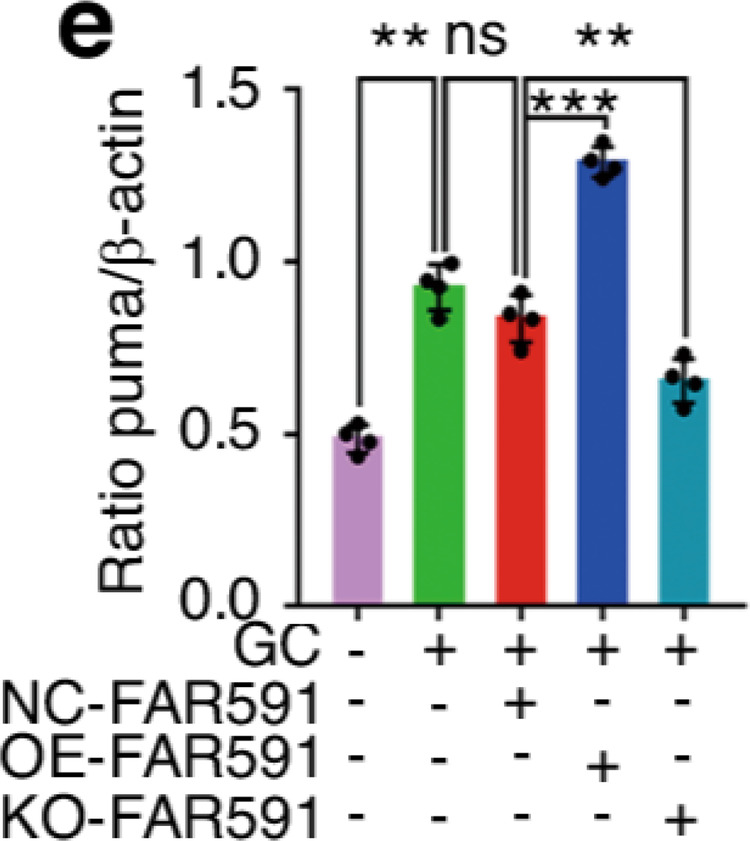


The original figure 4d was:
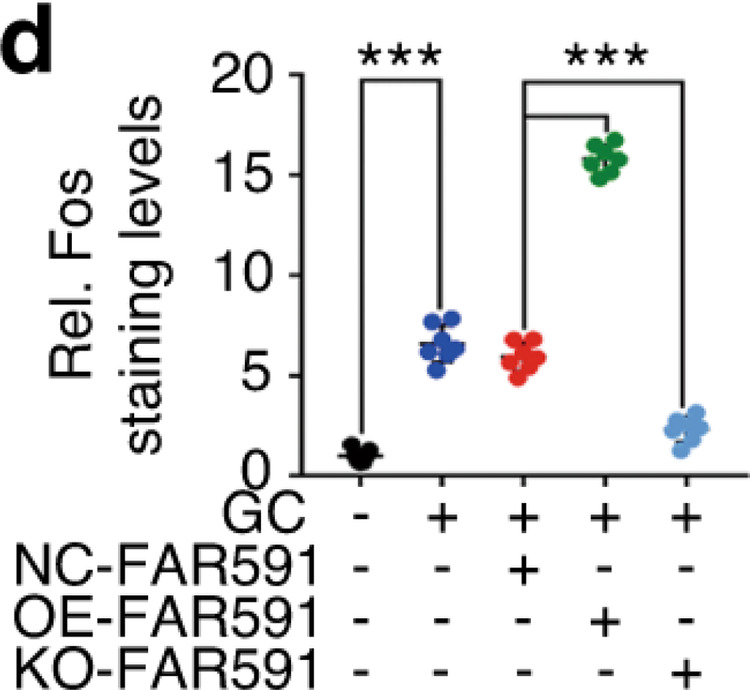


The correct figure 4d should read:
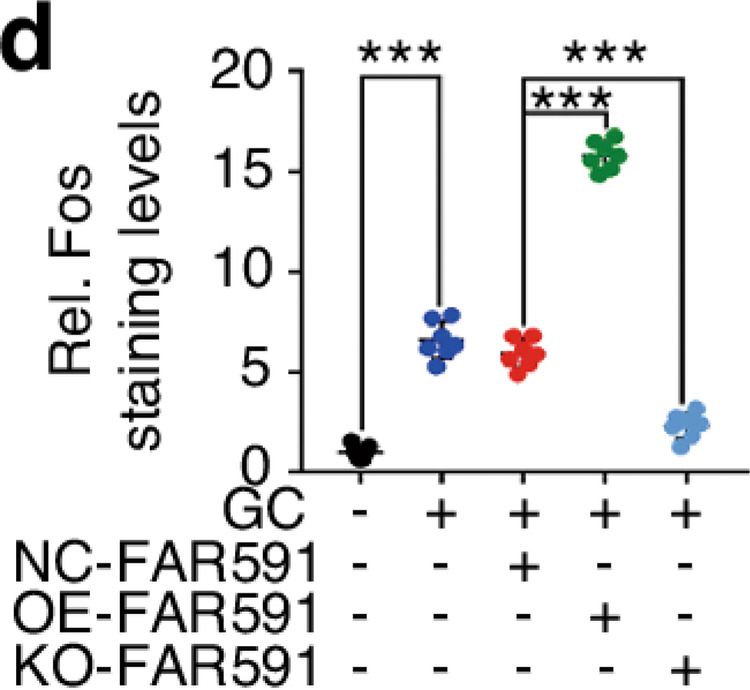


The original figure 6d was:
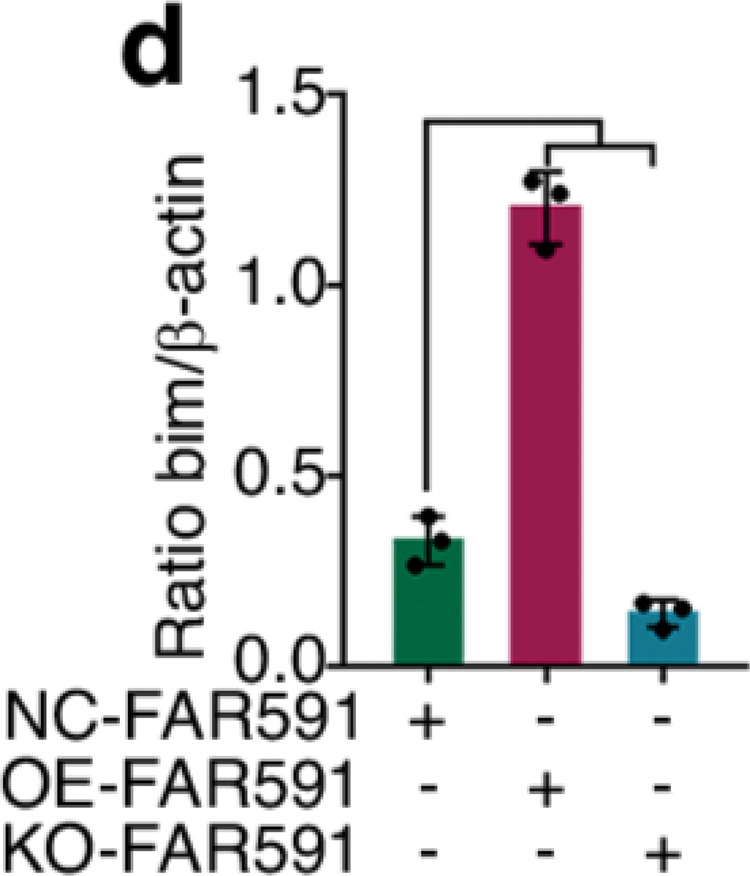


The correct figure 6d should read:
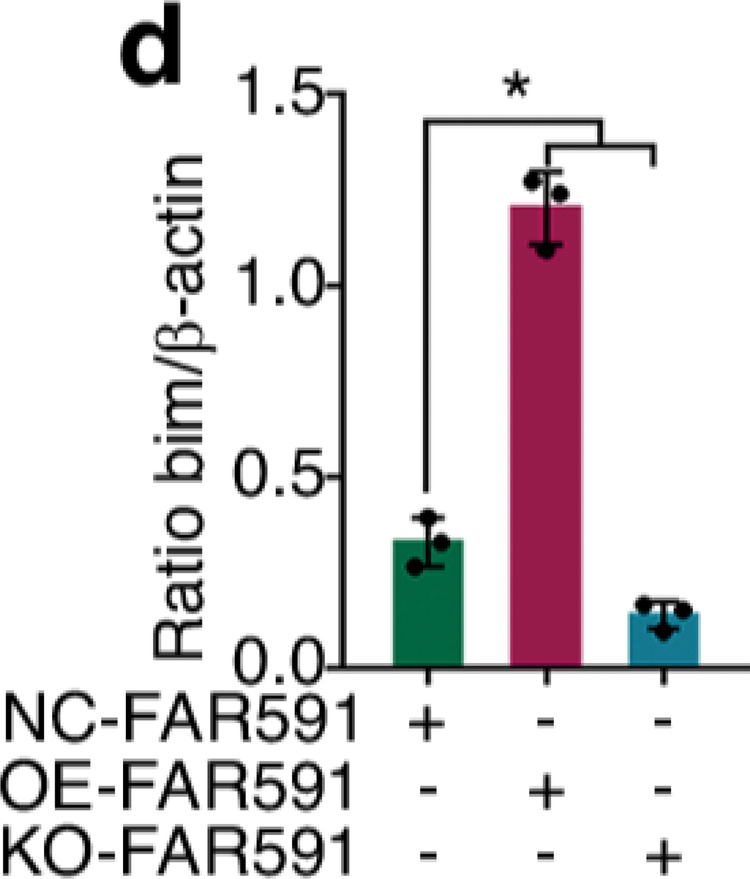


The original figure 8m was:
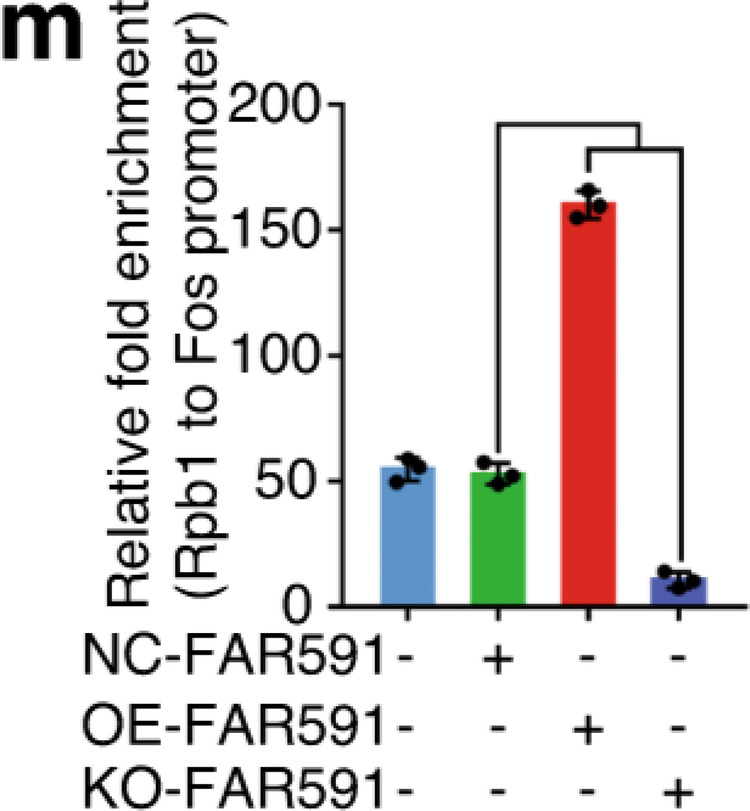


The correct figure 8m should read:
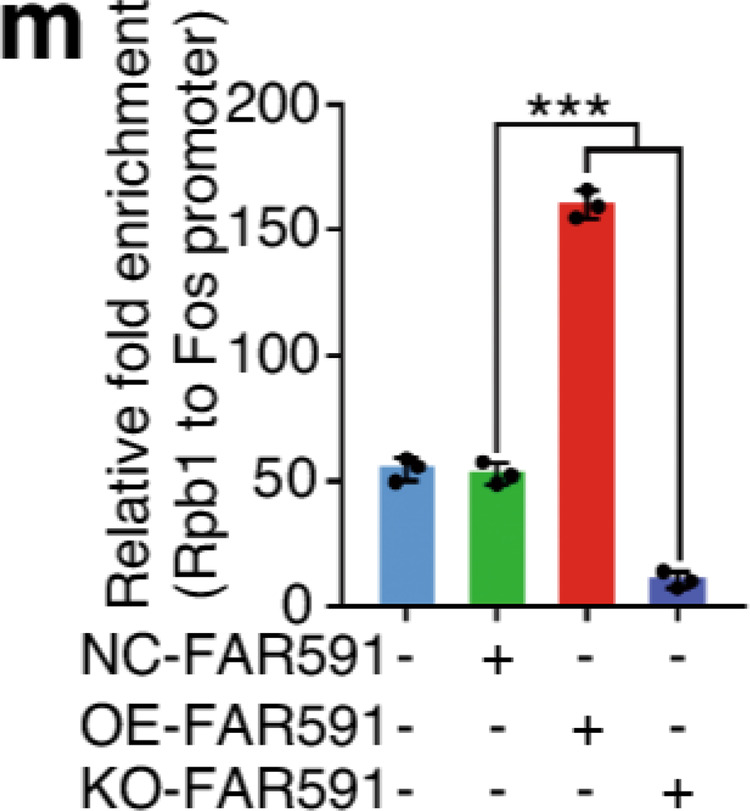


The original article^1^ has been updated.
