# Supplementary material for: FAR591 promotes the pathogenesis and progression of SONFH by regulating Fos expression to mediate the apoptosis of bone microvascular endothelial cells
Source: Bone Res. 2023 May 22;11:27. doi: 10.1038/s41413-023-00259-8 (PMC10203311; doi:10.1038/s41413-023-00259-8)
Supplement: Supplementary file 1 — Supplementary Materials [file 41413_2023_259_MOESM1_ESM.pdf]

Supplementary material

1. GC induced apoptosis of BMECs and inhibited its tubular formation

BMECs are the main functional cells of bone angiogenesis. We successfully isolated and cultured BMECs from femoral head tissue with a positive rate of over 99% for vWF and CD31 (Supplementary Fig. 1A and B) and successfully induced tubulogenesis (Supplementary Fig. 1C). BMEC apoptosis was induced by hydrocortisone (HC) at different concentrations. HC is the active form of GC in cells, and can directly bind to the glucocorticoid receptor. Compared to the control group (0 mg/mL), HC at a concentration of 0.1 mg/mL had no significant effect on the tubulogenesis, apoptosis and activity of BMECs (Supplementary Fig. 1D–J). When the concentration of HC was 0.2 mg/mL, the tubulogenesis of BMECs was inhibited, cleaved-CASP3 expression was up-regulated, BMEC apoptosis was increased, and cell viability was decreased, all with statistical significance (all $P < 0.05$; Supplementary Fig. 1D–J). When the concentration of HC was 0.3 mg/mL, it significantly inhibited the tubulogenesis of BMECs, significantly up-regulated the expression of cleaved-CASP3, further increased the apoptosis rate of BMECs, and significantly reduced the cell viability (Supplementary Fig. 1D–J). When the HC concentration was 0.4 mg/mL, numerous BMECs were apoptotic and could not form tubules (Supplementary Fig. 1D–G). These results showed that HC at 0.2 mg/mL could induce BMEC apoptosis and inhibit tubulogenesis.

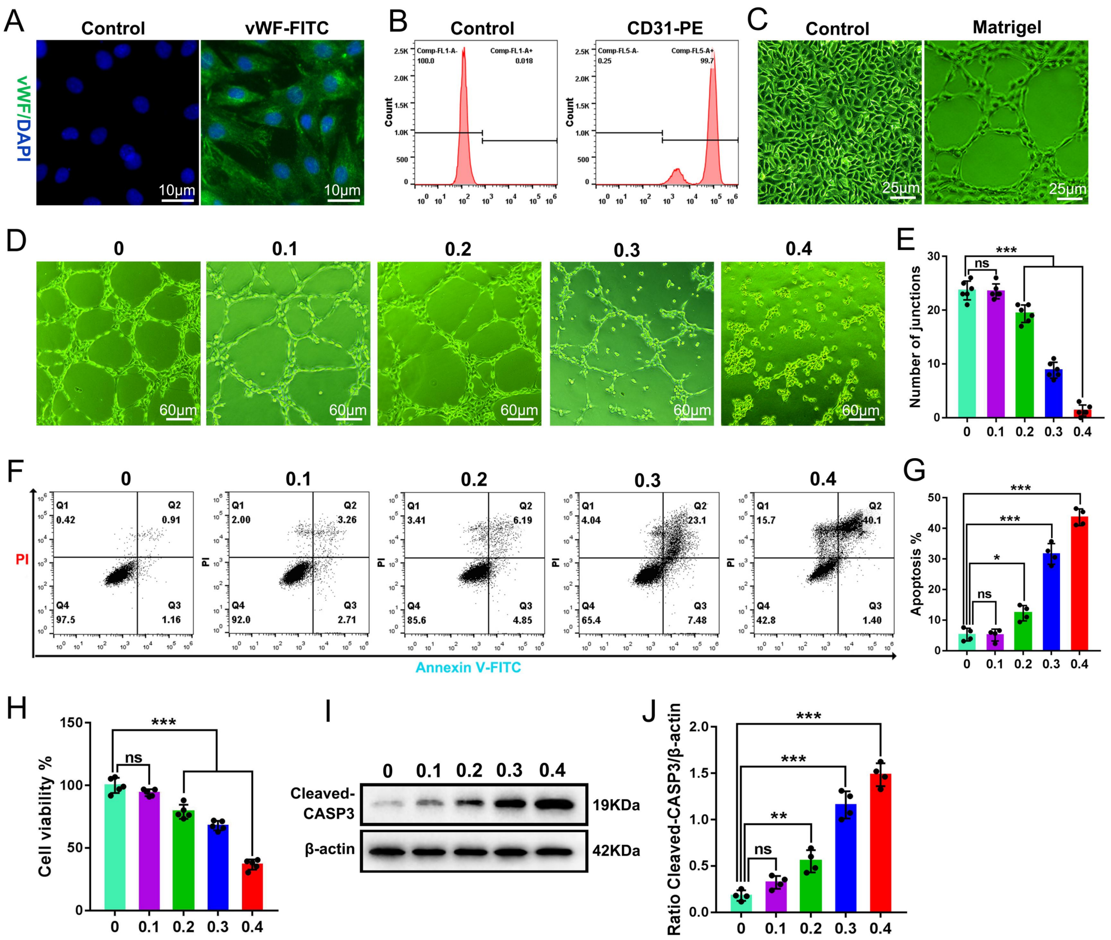

Supplementary Figure 1. GC induced apoptosis of BMECs and inhibited their tubular formation. Identification of BMECs: A Immunofluorescence detection of BMEC-specific antigens vWF (n = 5). **B** Flow cytometry Detection of BMEC surface antigens CD31 (n = 5). **C** BMEC tube formation (n = 5). **HC induced apoptosis and inhibited tube formation of BMECs: D–E** BMEC tube formation (n = 6). **F–G** Apoptosis of BMECs was detected by flow cytometry (n = 4). **H** The activity of BMECs was detected by CCK-8 (n = 5). **I–J** The expression of cleaved-CASP3 was detected by western blotting (n = 4). In (**E**, **G–H**, **J**), data are presented as means ± standard deviations (SDs); statistical significance was calculated by one-way ANOVA with Tukey's *post hoc* tests; $^{*}P < 0.05$, $^{**}P < 0.01$, $^{***}P < 0.001$. Abbreviations: glucocorticoid (GC), bone microvascular endothelial cells (BMECs), von Willebrand factor (vWF), fluoresceine isothiocyanate (FITC), hydrocortisone (HC), phycoerythrin (PE), propidium iodide (PI), caspase-3 (CASP-3).

2. GC induced apoptosis of OBs and inhibited osteogenesis

OBs are the main functional cell of bone regeneration. We successfully isolated and cultured OBs from femoral head tissue, and the OBs were positive for both alkaline phosphatase staining and calcium nodular staining (Supplementary Fig. 2A). OB apoptosis was induced by HC at different concentrations. Compared to the control group (0 mg/mL), HC at 0.1–0.2 mg/mL had no significant effect on OB osteogenesis, apoptosis, or viability (Supplementary Fig. 2B–H). When the HC concentration was 0.3 mg/mL, OB osteogenic activity was inhibited, cleaved-CASP3 expression was up-regulated, OB apoptosis was increased, and cell viability was decreased, all with statistical significance (all $P < 0.05$; Supplementary Fig. 2B–H). When the HC concentration was > 0.4 mg/mL, OB osteogenic activity was significantly inhibited, cleaved-CASP3 expression and OB apoptosis were significantly increased, and cell viability was further reduced (Supplementary Fig. 2B–H). These results showed that a higher HC concentration of 0.3 mg/mL was required to induce OB apoptosis and inhibit osteogenesis.

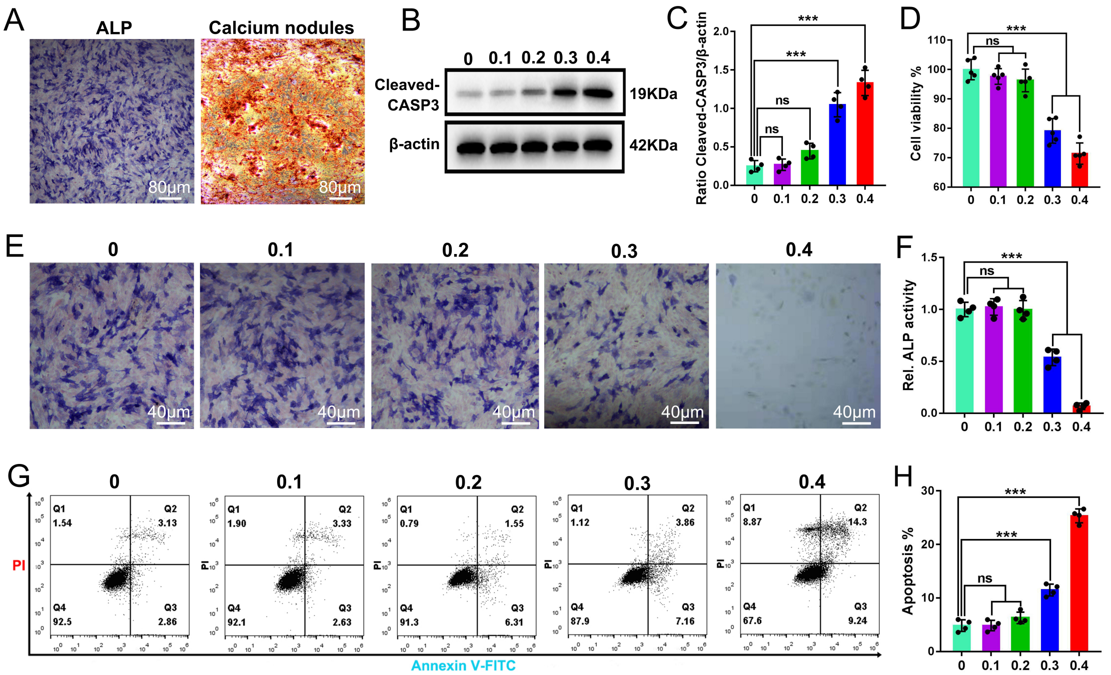

Supplementary Figure 2. GC induced OB apoptosis and inhibited osteogenesis. Identification of OB: A Alkaline phosphatase staining and calcium nodular staining (n = 5). **HC induced OB apoptosis and inhibited osteogenesis: B–C** The expression of cleaved-CASP3 was detected by western blot (n = 4). **D** The activity of OB was detected by CCK-8 (n = 5). **E–F** The osteogenic activity of OBs was detected by ALP staining (n = 4). **G–H** Apoptosis of OBs was detected by flow cytometry (n = 4). In (**C–D**, **F**, **H**), data are presented as the means ± SDs; statistical significance was calculated by one-way ANOVA with Tukey's *post hoc* tests; $^{*}P < 0.05$, $^{**}P < 0.01$, $^{***}P < 0.001$. Abbreviations: osteoblast (OB), alkaline phosphatase (ALP).

3. TRAP staining to evaluate the osteoclast number and bone resorption activity at 2 and 4 weeks after establishment of the SONFH model by GC

The osteoclast number and bone resorption activity were evaluated via TRAP staining at 2 and 4 weeks after establishment of the SONFH model by GC. The results showed that there was no significant change in the number of osteoclasts and bone resorption activity 2 weeks after injection of MP (Supplementary Fig. 3A–D); however, there was an increase in the number and bone resorption activity of osteoclasts 4 weeks after injection of MP (Supplementary Fig. 3E–H), which was consistent with the micro-CT results.

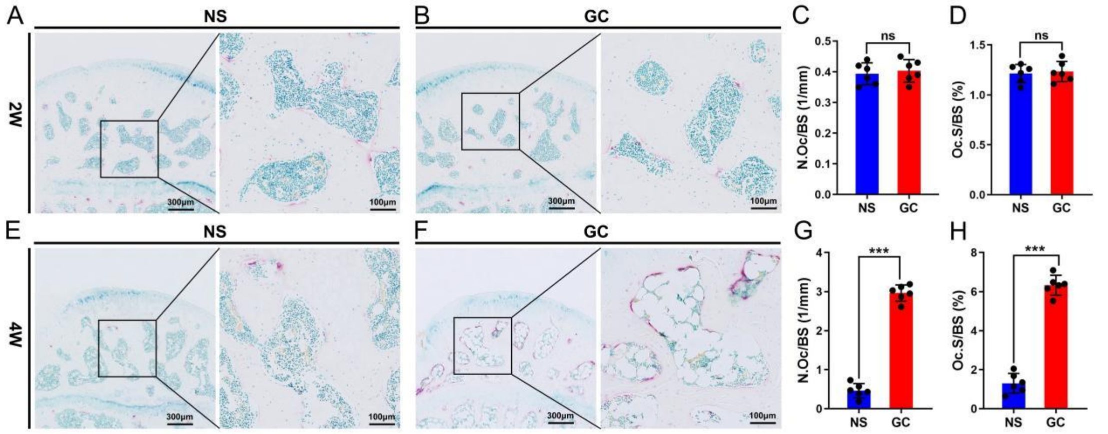

Supplementary Figure 3. TRAP staining to evaluate the osteoclast number and bone resorption activity at 2 and 4 weeks after establishment of the SONFH model by GC. Two weeks after MP gluteal injection (MP is a GC used for intramuscular injection): **A–B** Representative images of TRAP-stained sections of the femoral head (n = 6). **C** number of osteoclasts per bone surface (n = 6). **D** Osteoclast surface per bone surface (n = 6). Four weeks after MP injection: **E-F** Representative images of TRAP-stained sections of the femoral head (n = 6). **G** Number of osteoclasts per bone surface (n = 6). **H** Osteoclast surface per bone surface (n = 6). In (**C–D**, **G–H**), data are presented as the means ± SDs; statistical significance was calculated by Student's t-test; $^{*}P < 0.05$, $^{**}P < 0.01$, $^{***}P < 0.001$. Abbreviations: normal saline (NS), glucocorticoid (GC), methylprednisolone (MP), tartrate resistant acid phosphatase (TRAP), number of osteoclasts per bone surface (N.Oc/BS), osteoclast surface per bone surface (Oc.S/BS).

4. qPCR and bioinformatics analysis of lncRNA microarray data to screen FAR591

We used GC to induce apoptosis of BMECs, and screened differentially expressed genes by lncRNA/mRNA microarray. For the microarray data, we randomly selected eight genes (AABR07065091.1, LOC100909396, LOC100912629, LOC102548045, LOC102553043, LOC102554532, LOC103693247, AABR07020987.1) from the differentially expressed genes and validated them in the model of GC-induced BMEC apoptosis. The results showed that except for AABR07020987.1, the expression of the other seven genes were consistent with the microarray data (Supplementary Fig. 4A–H), which confirmed the accuracy of the microarray data. We selected the core mRNAs of the mitochondrial apoptosis pathway from the differentially expressed genes, and screened out four lncRNAs (LOC102550111, ENSRNOT00000088059.1, LOC100909396, LOC103693421) by mRNA-lncRNA co-expression analysis, which were found to be co-expressed with the above-mentioned core mRNAs in the model of BMEC apoptosis induced by different concentrations of GC (Supplementary Fig. 4I). We next screened out four lncRNAs (ENSRNOT00000088059.1, LOC102548045, LOC102555660, LOC102555576) through the adjacent gene screening (< 200 kb), which were adjacent to the above-mentioned core mRNAs and expression could be induced by GC (Supplementary Fig. 4J). ENSRNOT00000088059.1 is not only adjacent to the core mRNAs of mitochondrial apoptosis pathway, but also has a co-expression relationship with the core mRNAs. Therefore, ENSRNOT00000088059.1 may be a candidate lncRNA related to apoptosis (Supplementary Fig. 4K).

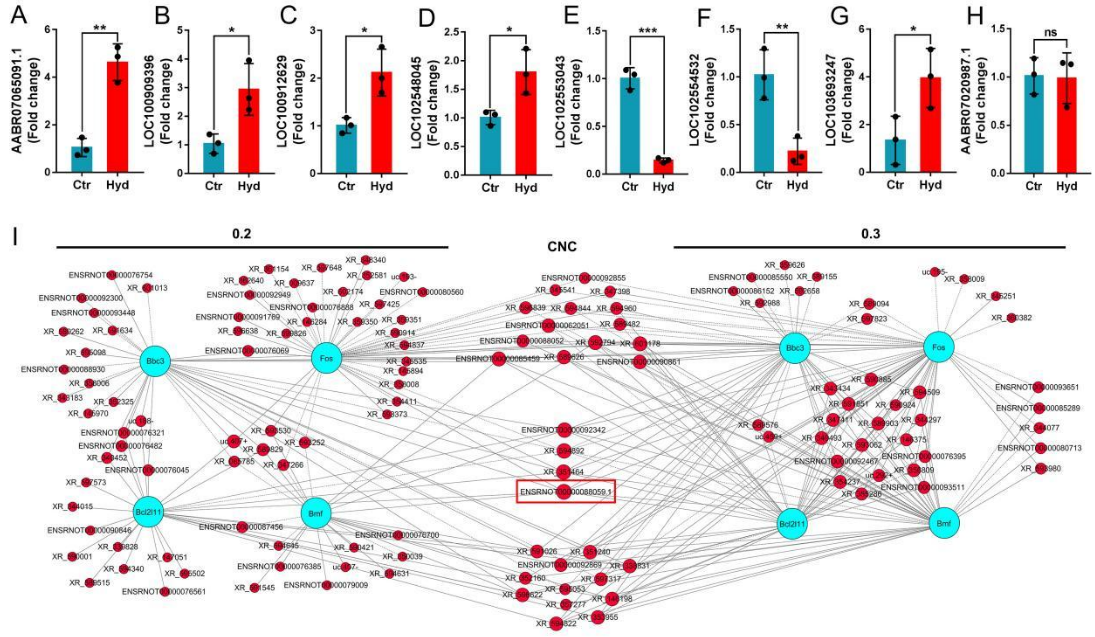

J

GC-dependent core mRNA of the mitochondrial apoptotic pathway	LncRNA adjacent to mRNA	Distance	LncRNA			
			Regulation	Fold Change	FDR	P-value
Fos	ENSRNOT00000088059.1	< 200 kb	Up	6.10	0.00	0.00
	LOC102555660	< 200 kb	Down	4.20	0.02	0.00
Bcl2l11	LOC102555576	< 200 kb	Down	1.24	0.46	0.22
	LOC120101657	< 200 kb	—	—	—	—
Bbc3	LOC120098274	< 200 kb	—	—	—	—
Bmf	LOC102548045	< 200 kb	Up	3.11	0.06	0.00

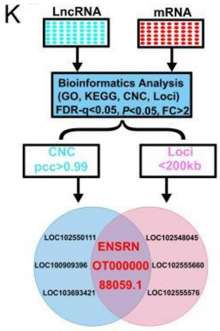

Supplementary Figure 4. Validation of lncRNA microarray data and screening of FAR591. qPCR validation of lncRNA microarray data: A–H qPCR detection of AABR07065091.1, LOC100909396, LOC100912629, LOC102548045, LOC102553043, LOC102554532, LOC103693247, and AABR07020987.1 (n = 3). **Screening of FAR591: I** Co-expression analysis of the mitochondrial apoptosis pathway core mRNA-lncRNA. **J** Screening of adjacent genes of the core mRNA of the mitochondrial apoptosis pathway. **K** Schematic diagram of FAR591 screening. In (**A–H**), data are presented as the means ± SDs; statistical significance was calculated by Student's t-test; $^{*}P < 0.05$, $^{**}P < 0.01$, $^{***}P < 0.001$. Abbreviations: long noncoding RNA (lncRNA), Fos-associated lincRNA ENSRNOT00000088059.1 (FAR591), coding/non-coding gene co-expression (CNC), Bcl-2 interacting mediator of cell death (Bim), Bcl-2 modifying factor (Bmf), P53 upregulated modulator of apoptosis (Puma), false discovery rate (FDR), gene ontology (GO), Kyoto Encyclopedia of Genes and Genomes (KEGG), locus (Loci).

5. TRAP staining to evaluate the osteoclast number and bone resorption activity in the FAR591 functional experiment

The osteoclast number and bone resorption activity were evaluated via TRAP staining in the in vivo FAR591 functional experiment. The results showed that knockout of FAR591 reduced the number of osteoclasts and bone resorption activity, while overexpression of FAR591 significantly increased the number of osteoclasts and bone resorption activity (Supplementary Fig. 5A–C), which further supported the micro-CT results.

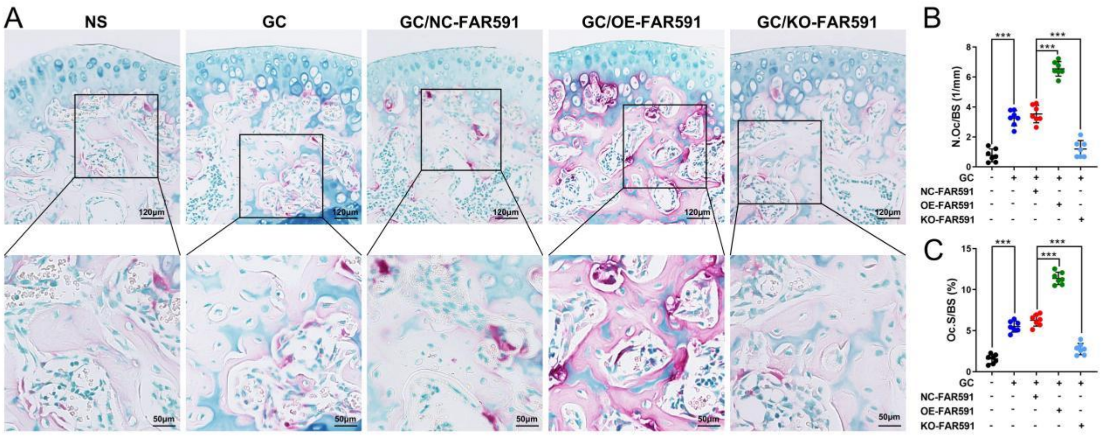

Supplementary Figure 5. TRAP staining to evaluate the osteoclast number and bone resorption activity in the functional experiment of FAR591. A Representative images of TRAP-stained sections of the femoral head (n = 7). **B** Number of osteoclasts per bone surface (n = 7). **C** Osteoclast surface per bone surface (n = 7). In (**B–C**), data are presented as the means ± SDs; statistical significance was calculated by one-way ANOVA with Tukey's *post hoc* tests; $^{*}P < 0.05$, $^{**}P < 0.01$, $^{***}P < 0.001$. Abbreviations: negative control (NC), overexpression (OE), knockout (KO).

6. FAR591 regulated the expression of Fos mRNA and protein

FAR591 was overexpressed or knocked out in BMECs (OE-FAR591 or KO-FAR591), and the lentiviral empty vector was used as a negative control (NC-FAR591). The expression of Fos mRNA and protein was detected by qPCR and immunoblotting, and the results showed that overexpression of FAR591 up-regulated the expression levels of Fos mRNA and protein (Supplementary Fig. 6A–C). In contrast, knockout of FAR591 down-regulated the expression of Fos mRNA and protein (Supplementary Fig. 6D–F).

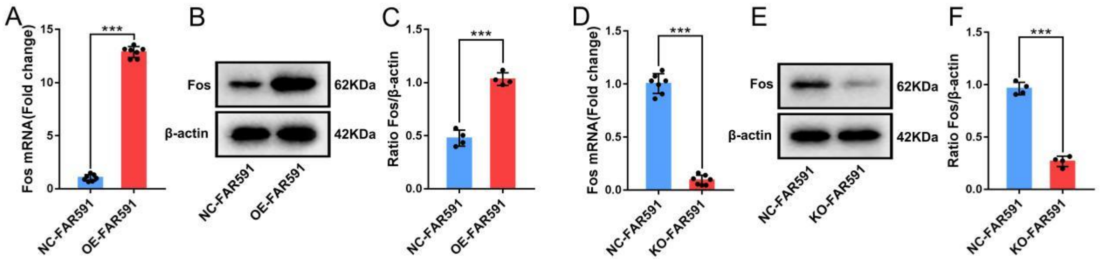

Supplementary Figure 6. FAR591 regulated the expression of Fos. Overexpression of FAR591: A qPCR detection of Fos mRNA expression (n = 7). **B–C** Western blot detection of Fos protein expression (n = 4). **Knockout of FAR591: D** qPCR detection of Fos mRNA expression (n = 7). **E–F** Western blot detection of Fos protein expression (n = 4). In (**A**, **C–D**, **F**), data are presented as the means ± SDs; statistical significance was calculated by Student's t-test; $^{*}P < 0.05$, $^{**}P < 0.01$, $^{***}P < 0.001$.

7. ChIRP-Seq/MS detection of cis-acting elements and trans-acting factors interacting with FAR591

We used chromatin isolation by RNA purification (ChIRP) technology to isolate cis-acting elements and trans-acting factors interacting with FAR591 in BMECs. The high-throughput sequencing (Seq) results showed that a total of 80523 differentially enriched peaks of FAR591 were detected in genomic DNA ($P < 10^{-5}$; Supplementary Fig. 7A); 57.95% of these differential enrichment peaks were located in intergenes, 1.19% were located in exons, 25.51% were located in introns, 11.00% were located in the upstream region of the gene, and 4.35% were located in the gene promoter region (Supplementary Fig. 7B), among which, the enrichment peaks in the promoter region were mainly distributed near the transcriptional start site (TSS ± 5.00 kb; Supplementary Fig. 7C). Mass spectrometry (MS) analysis showed that FAR591 could specifically bind to 26 proteins (Supplementary Fig. 7D–E). We isolated the RNA interacting with TAF15 using the RNA binding protein immunoprecipitation (RIP) technique and found that FAR591 was significantly enriched in the complex precipitated by the TAF15 antibody (Supplementary Fig. 7F). We used RPISeq and catRAPID software to analyze the interaction site between TAF15 and FAR591. The results showed that GGUG was the motif sequence of the RNA recognized by TAF15 (Supplementary Fig. 7G), and there may be two binding sites on FAR591, both located at the 5' end of FAR591 (Supplementary Fig. 7H–I).

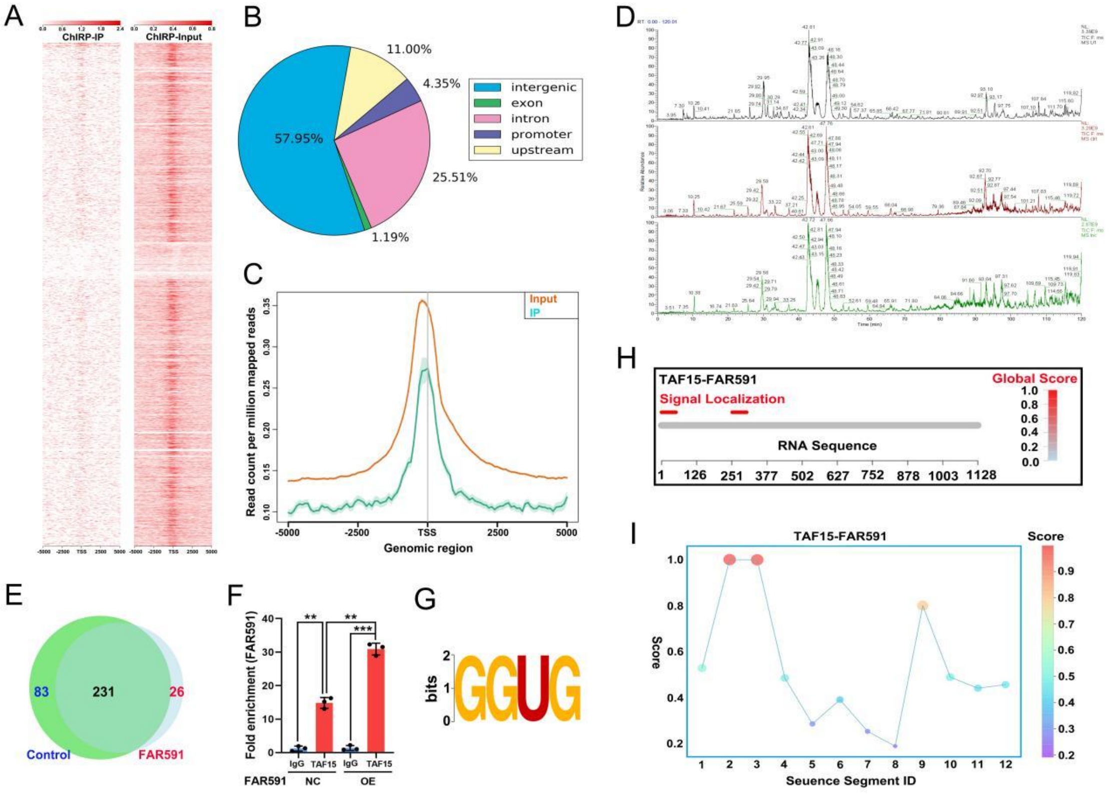

Supplementary Figure 7. ChIRP-Seq/MS detection of cis-acting elements and trans-acting factors interacting with FAR591. A Heat map of Seq results (n = 3). **B** Pie chart of peak position distribution. **C** Plots of promoter region sequencing coverage. **D** Mass spectrum peaks of LC-MS/MS analysis (n = 3). **E** MS identified 26 proteins specifically binding to FAR591. **F** RIP detected the interaction of FAR591 with TAF15 (n = 3). **G** TAF15 recognizes motif sequences of RNA. **H–I** RPISeq and catRAPID predicted the binding sites of TAF15 on FAR591. In (**F**), data are presented as the means ± SDs; statistical significance was calculated by one-way ANOVA with Tukey's *post hoc* tests; $^{*}P < 0.05$, $^{**}P < 0.01$, $^{***}P < 0.001$. Abbreviations: chromatin isolation by RNA purification (ChIRP), RNA binding protein immunoprecipitation (RIP), liquid chromatography (LC), mass spectrometry (MS).

8. Tables

Table 1 FAR591 probe sequence

Probe Number	Probe Sequence
2103171A-P1	TCAAATCAGCTGGTGGTGAG
2103171A-P2	ACTACTGTGATGATGCCTAC
2103171A-P3	TGCCAAGCTTGACAAACTGG
2103171A-P4	AACCTTCCCGAGCGTTTCAT
2103171A-P5	AGGCGTTGGGGACACTTTAC
2103171A-P6	TTTGTACAGATGTGCCGGGT
2103171A-P7	TCTAGCCACTAAAGCACAGCG
2103171A-P8	TGACATGACAGGTATGAGCCA
2103171A-P9	ATAAGCCACAGAAAGAAGCCAGC
2103171A-P10	AAGCCAACCTGGAGGAAAGG

Table 2 Primers for the (−500 to +1000) region of Fos promoter

Primer Name	Primer Sequence
P1(1-192) F	GACCCCCAGTCTCATCCC
P1(1-192) R	CCTGTGTAAAGGAGGGAGGGATT
P2(135-311) F	TCAGAGTTGGCTGCAGCCG
P2(135-311) R	ATTGGGATCTTAGAAGGTCTCCTGGA
P3(255-449) F	GAACCATCCCCGAAATCCTACATGC
P3(255-449) R	TACGTCATGAGCGGAACAGAGAAACT
P4(392-580) F	CAGAGGGCATTCGGGAGC
P4(392-580) R	GTGGGAGCGCGGTCACT
P5(534-708) F	TGGATAGAGCCGGCGGAGC
P5(534-708) R	CTACTGCAGCGGGAGGATGAC
P6(668-864) F	TTCAACGCGGACTACGAGGC
P6(668-864) R	AGTCACACCCCAATGTGCC
P7(806-982) F	TTGTGCAGTCGCCAGGTCC

P7(806-982) R	TCTTAGTATGACAAGTGTGCACGCG
P8(930-1106) F	TAGCCTGGGAACCCAGGACT
P8(930-1106) R	AGGCTCTAGTTAGCGAGTCTTTGC
P9(1054-1247) F	TGAGATCAGCCGGGGCC
P9(1054-1247) R	TGCTCCGGAGTCTCCAGAATG
P10(1187-1370) F	AGCCGGTGTGTAAGGCAG
P10(1187-1370) R	TGAATTCCGCAGCTCAGCCTT
P11(1326-1500) F	AACGCAGCAGTAGGATGGAGG
P11(1326-1500) R	AGGTGGAGCAAGTGGCC

Table 3 Primers for the (−1000 to +1000) region of Fos promoter

Primer Name	Primer Sequence
P1(1-171) F	ATTCAGGTCCCCACCTCCC
P1(1-171) R	CGGATAAACATTGTGCAAAACCAC
P2(123-295) F	CAAACGACCCCTTCAGGCAT
P2(123-295) R	ACCCGCTTTACCTACAATACTGA
P3(242-430) F	CGTCCTTTAAAACACGAATGTATGA
P3(242-430) R	CAGCCCTAAAGGCTCCAAGT
P4(370-559) F	AGAGAGGTCTGATGTGGGCTA
P4(370-559) R	ATTCGCACCTGATTCAATGTGTA
P5(501-692) F	GACCCCCAGTCTCATCCC
P5(501-692) R	CCTGTGTAAAGGAGGGAGGGATT
P6(635-811) F	TCAGAGTTGGCTGCAGCCG
P6(635-811) R	ATTGGGATCTTAGAAGGTCTCCTGGA
P7(755-949) F	GAACCATCCCCGAAATCCTACATGC
P7(755-949) R	TACGTCATGAGCGGAACAGAGAAACT
P8(892-1080) F	CAGAGGGCATTCGGGAGC
P8(892-1080) R	GTGGGAGCGCGGTCACT
P9(1034-1208) F	TGGATAGAGCCGGCGGAGC

P9(1034-1208) R	CTACTGCAGCGGGAGGATGAC
P10(1168-1364) F	TTCAACGCGGACTACGAGGC
P10(1168-1364) R	AGTCACACCCCAATGTGCC
P11(1306-1482) F	TTGTGCAGTCGCCAGGTCC
P11(1306-1482) R	TCTTAGTATGACAAGTGTGCACGCG
P12(1430-1606) F	TAGCCTGGGAACCCAGGACT
P12(1430-1606) R	AGGCTCTAGTTAGCGAGTCTTTGC
P13(1554-1747) F	TGAGATCAGCCGGGGCC
P13(1554-1747) R	TGCTCCGGAGTCTCCAGAATG
P14(1687-1870) F	AGCCGGTGTGTAAGGCAG
P14(1687-1870) R	TGAATTCCGCAGCTCAGCCTT
P15(1826-2000) F	AACGCAGCAGTAGGATGGAGG
P15(1826-2000) R	AGGTGGAGCAAGTGGCC

Table 4 cDNA synthesis system

Reagent Category	Reagent Dosage
RNA (100 ng/μL)	100.00 μL
Oligo (dT) (0.5 μg/μL)	10.00 μL
RNase free ddH_2O	10.00 μL
65°C 5 min, ice bath 30 s	
5× Reaction buffer	40.00 μL
RNase inhibitor (40 U/μL)	10.00 μL
dNTP mix (10 mmol/L)	20.00 μL
M-MuLV RT (200 U/μL)	10.00 μL
42°C 30 min, 70°C 10 min	

Table 5 qPCR reaction system

Reagent Category	Reagent Dosage
Template DNA	1.00 μL

Primer-F	1.00 μL
Primer-R	1.00 μL
DNF buffer	2.00 μL
PCR-grade water	5.00 μL
2× SG Fast Qpcr Master Mix (High Rox)	10.00 μL
95°C 3 min; 95°C 3 s, 60°C 30 s, 40 cycles	

Table 6 Primer sequences

Primer Name	Primer Sequence
FAR591-F	TCCTCTTCCTTCTGACTGCTCTT
FAR591-R	TGTGATGATGCCTACGGGACT
LOC102548045-F	AGGCACCGTGACCACAACAATC
LOC102548045-R	CCACCATGCTCCCATCAGGATAAC
AABR07020987.1-F	TCCTGCCTCCTCCAACCTTCTATC
AABR07020987.1-R	ACTGTGCCAAGACATCTGCCATAC
LOC100912629-F	CAATGGGAAGCTCCTGACGC
LOC100912629-R	GGTCCGCCCTCAACAAACTC
LOC103693247-F	GTTATTTGTTCCTTGGGCCATGC
LOC103693247-R	AGCCATCATTCTCATCGCATGTT
LOC100909396-F	TCGAAGGAAGAGAACAGCCAGGAG
LOC100909396-R	CAGCATGGTATGGCACTCTGAAGG
LOC102553043-F	GGTCGCAGGTTCCAGGTTGTG
LOC102553043-R	GGTTCTGTTGGCTGAGGCTCAC
LOC102554532-F	CTGTCCCTCCTGTCTTAGGCTGAC
LOC102554532-R	GGCTGGGAAATGATGGTCTGGTTC

Table 7 Primers for FAR591

Primer Name	Primer Sequence
FAR591-F	CCTGTCATGTCACTATGTAGGT

FAR591-R	GGTGAGGAGGCACCAATCTT

Table 8 PCR reaction system

Reagent Category	Reagent Dosage
ddH_2O	1.00 μL
FAR591-F (10 μM)	1.00 μL
FAR591-R (10 μM)	1.00 μL
cDNA templates	2.00 μL
Platinum® PCR SuperMix, High Fidelity	45.00 μL
94°C 2 min; 94°C 30 s, 62°C 30 s, 68°C 1 min, 40 cycles	

Table 9 Primers for 5′ RACE

Primer Name	Primer Sequence
FAR591-R1	CAGCCTGCAAGGTTGTTGCTGACTTGGT
FAR591-R2	CGCCACTCCAGGTGGATAATGAAGTACAG

Table 10 Primers for 3′ RACE

Primer Name	Primer Sequence
FAR591-F1	CGGCTAGTAAGGAACACCAAGTCAGCAACA
FAR591-F2	CCCTGCTCTGCCAAGGCCAAGGTGTCA

Table 11 First round of the 5′ RACE reaction system

Reagent Category	Reagent Dosage
5′ RACE templates	0.50 μL
5′ GeneRacer outer primer (10 μM)	1.00 μL
FAR591-R1 (10 μM)	1.00 μL
Platinum PCR Supermix High Fidelity (Invitrogen)	22.50 μL

94°C 2 min; 94°C 30 s, 72°C 30 s, 5 cycles; 94°C 30 s, 70°C 30 s, 5 cycles; 94°C 30 s, 66°C 30 s, 25 cycles	

Table 12 Second round of the 5′RACE reaction system

Reagent Category	Reagent Dosage
Product of the first round of 5′ RACE	0.50 μL
5′ GeneRacer inner primer (10 μM)	1.00 μL
FAR591-R2 (10 μM)	1.00 μL
Platinum PCR Supermix High Fidelity (Invitrogen)	22.50 μL
94°C 2 min; 94°C 30 s, 66°C 30 s, 30 cycles	

Table 13 First round of the 3′RACE reaction system

Reagent Category	Reagent Dosage
3′ RACE templates	0.50 μL
3′ GeneRacer outer primer (10 μM)	1.00 μL
FAR591-F1 (10 μM)	1.00 μL
Platinum PCR Supermix High Fidelity (Invitrogen)	22.50 μL
94°C 2 min; 94°C 30 s, 72°C 30 s, 5 cycles; 94°C 30 s, 70°C 30 s, 5 cycles; 94°C 30 s, 66°C 30 s, 25 cycles	

Table 14 Second round of the 3′RACE reaction system

Reagent Category	Reagent Dosage
Product of the first round of 3′ RACE	0.50 μL
3′ GeneRacer Inner primer (10 μM)	1.00 μL
FAR591-F2 (10 μM)	1.00 μL
Platinum PCR Supermix High Fidelity (Invitrogen)	22.50 μL
94°C 2 min; 94°C 30 s, 66°C 30 s, 30 cycles	

Table 15 Nested PCR primers for FAR591

Primer Name	Primer Sequence
Peripheral primer-F	GGAGGCCATCTCTGGAGACCATG
Peripheral primer-R	AAGCTGGGACAGCAGAGTAAGAC
Internal primer-F	AATGGTGATTCACACTCCAACAC
Internal primer-R	AACTCCGAATTGTGCAAAGCCAC

Table 16 Peripheral PCR reaction system

Reagent Category	Reagent Dosage
2× Dream taq green PCR master mix	12.50 μL
Peripheral primer-F (10 μM/L)	1.00 μL
Peripheral primer-R (10 μM/L)	1.00 μL
DNA (cell samples, 10 ng/μL)	10.50 μL
95°C 5 min; 95°C 30 s, 63°C 30 s, 72°C 1 min, 40 cycles; 72°C 10 min	

Table 17 Internal PCR reaction system

Reagent Category	Reagent Dosage
2× Dream taq green PCR master mix	12.50 μL
Internal primer-F (10 μmol/L)	1.00 μL
Internal primer-R (10 μmol/L)	1.00 μL
DNA (peripheral PCR products, diluted 800×)	10.50 μL
95°C 5 min; 95°C 30 s, 61°C 30 s, 72°C 1 min, 40 cycles; 72°C 10 min	

Table 18 PCR primers for Fos

Primer Name	Primer Sequence
Fos-F	GGTTTCAACGCGGACTACGA
Fos-R	TGGCACTAGAGACGGACAGA

Table 19 Formulation system for the 12% separation gel

Reagent Category	Reagent Dosage
ddH_2O	4.950 mL
30% Acrylamide (29:1)	6.000 mL
1.5 M Tris-Hcl (pH 8.8)	3.750 mL
10% SDS	0.150 mL
10% APS	0.150 mL
TEMED	0.015 mL

Table 20 Formulation system for the 5% separation gel

Reagent Category	Reagent Dosage
ddH_2O	4.104 mL
30% Acrylamide (29:1)	0.996 mL
1 M Tris-Hcl (PH 6.8)	0.750 mL
10% SDS	0.060 mL
10% APS	0.090 mL
TEMED	0.009 mL

Table 21 TdT incubation buffer system

Reagent Category	Reagent Dosage
ddH_2O	34.00 μL
5× Equilibration Buffer	10.00 μL
BrightRed Labeling Mix	5.00 μL
Recombinant TdT Enzyme	1.00 μL
